# Left Atrial trajectory impairment in Hypertrophic Cardiomyopathy disclosed by Geometric Morphometrics and Parallel Transport

**DOI:** 10.1038/srep34906

**Published:** 2016-10-07

**Authors:** Paolo Piras, Concetta Torromeo, Federica Re, Antonietta Evangelista, Stefano Gabriele, Giuseppe Esposito, Paola Nardinocchi, Luciano Teresi, Andrea Madeo, Claudia Chialastri, Michele Schiariti, Valerio Varano, Massimo Uguccioni, Paolo E. Puddu

**Affiliations:** 1 Dipartimento di Ingegneria Strutturale e Geotecnica, Sapienza Università di Roma, Roma, Italy; 2 Dipartimento di Scienze Cardiovascolari, Respiratorie, Nefrologiche, Anestesiologiche e Geriatriche, Sapienza Università di Roma, Roma, Italy; 3 Centro per le Cardiomiopatie Ospedale S. Camillo–Forlanini, Roma, Italy; 4 Ospedale San Giovanni Calibita Fatebenefratelli Isola Tiberina, Roma, Italy; 5 Dipartimento di Architettura, Università Roma Tre, Roma, Italy; 6 Dipartimento di Matematica e Fisica, LaMS–Modeling & Simulation Lab, Università Roma Tre, Roma, Italy

## Abstract

The analysis of full Left Atrium (LA) deformation and whole LA deformational trajectory in time has been poorly investigated and, to the best of our knowledge, seldom discussed in patients with Hypertrophic Cardiomyopathy. Therefore, we considered 22 patients with Hypertrophic Cardiomyopathy (HCM) and 46 healthy subjects, investigated them by three–dimensional Speckle Tracking Echocardiography, and studied the derived landmark clouds via Geometric Morphometrics with Parallel Transport. Trajectory shape and trajectory size were different in Controls versus HCM and their classification powers had high AUC (Area Under the Receiving Operator Characteristic Curve) and accuracy. The two trajectories were much different at the transition between LA conduit and booster pump functions. Full shape and deformation analyses with trajectory analysis enabled a straightforward perception of pathophysiological consequences of HCM condition on LA functioning. It might be worthwhile to apply these techniques to look for novel pathophysiological approaches that may better define atrio–ventricular interaction.

The number of contributions aimed at modelling heart mechanics and function using image-based data increases day by day. Both Cardiac Magnetic Resonance (MRI) and 2D or 3D Speckle Tracking Echocardiography (2DSTE, 3DSTE) are used for this purpose being 2DSTE and 3DSTE less invasive, faster and of easier access. Hypertrophic Cardiomyopathy (HCM) is one of the most prevalent heart diseases among those genetically determined^1^. Whereas HCM impact on Left Ventricle (LV) is largely studied^1^,^2^,^3^, investigations on Left Atrium (LA) are less common^4^,^5^,^6^,^7^,^8^,^9^,^10^. It has been found however, that LA in HCM presents several abnormalities when compared to healthy subjects^4^,^5^,^6^,^7^,^8^,^9^,^10^. Main findings concern increased LA dilation and reduced strain relative to Controls^4^,^5^,^6^,^7^,^8^,^9^,^10^.

Nevertheless, a full shape analysis of LA revolution in HCM is still lacking^10^. Typically, within the limits of 3DSTE–based studies, LA strains during cardiac revolution are acquired. However, longitudinal, circumferential, and radial strains as well as rotational parameters (twist, torsion, rotation) cannot return any information about the actual LA geometry. In fact, classic 3DSTE parameters compute at any time local shape changes as differences in relation to end–systolic state. Moreover, they are delivered as mean values on finite parts (segments) of the LA. On the contrary, managing the full shape information encrypted within landmarks’s coordinates measured by 3DSTE allows to appreciate the actual shape evolution, and to build a model able to *shaping* the motion trajectory, *i.e.* exploring its geometrical attributes. The first step towards this target is the extension in time of the concept of homology: as the anatomical homology is required to compare shapes, physiologically homologous times are needed in order to compare motion trajectories^11^,^12^. Moreover, these times have to be separately recognized within each individual and variables of interest (univariate such as 3DSTE parameters or multivariate such as shape) are interpolated at these times^11^,^12^. Following the above path, in this contribution we extend the acquired knowledge of LA functional impairment in HCM by performing a deformation analysis along with a trajectory analysis, *i.e.* an analysis of the shape of the motion path of LA, that we hypothesize different in HCM patients versus Controls.

## Results

**Figure 1:**
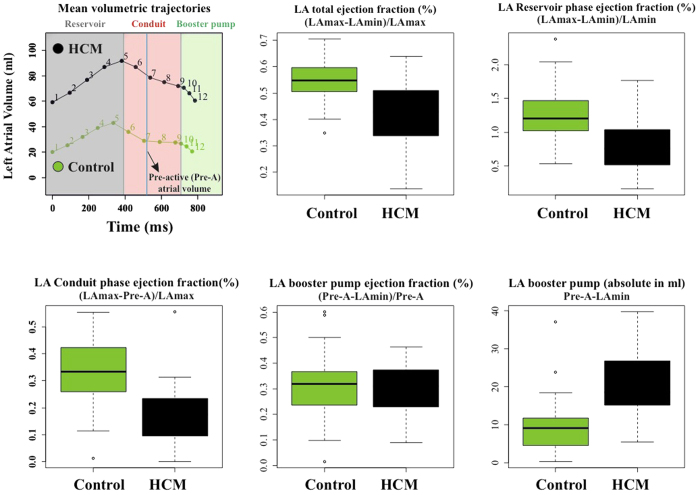
LA function volumetric indicators (evaluated as indicated by Hoit^14^).

**Figure 2:**
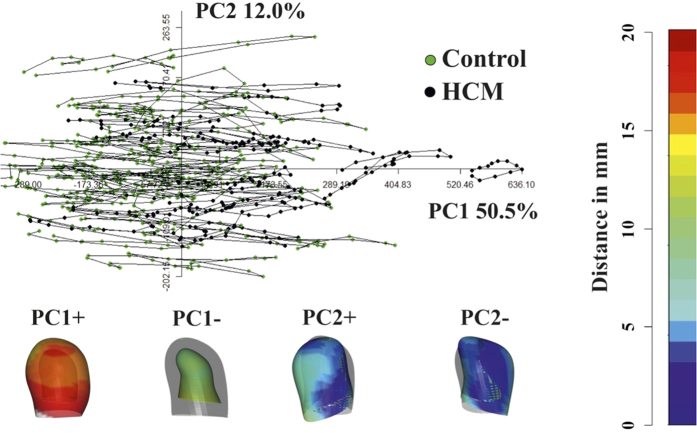
Classic GPA in SSS + PCA. This figure clearly shows that PCA performed after GPA mixes intra- and inter- individual variation, thus impeding to appreciate pure deformations occurring within individuals. Deformations associated to PC axes extremes are shown. These are relative to the GM that is shown in light grey together with coloured shapes corresponding to axes extremes. The colormap ranges from blue (min) to red (max) and refers to |*x*_*M*_ − *x*|, with *x*_*M*_ the position of a point in the GM and *x* its position at the specified axis extreme (positive or negative).

**Figure 3:**
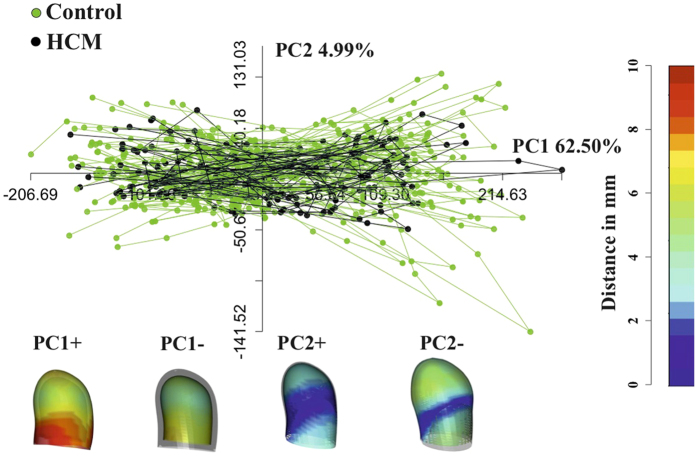
GPA in SSS + LS in SSS + PCA. The LS procedure centered on GM allows (by contrast to what seen in Fig. 2) the appreciation of pure deformation patterns without the noise due to inter-individual variation. Importantly, centering deformations on GM allows also to consider both systole and diastole as deformed states. Deformations relative to the GM (shown in light grey) are depicted. The colormap ranges from blue (min) to red (max) and refers to |*x*_*M*_ − *x*|, with *x*_*M*_ the position of a point in the GM and *x* its position at the specified axis extreme (positive or negative).

Figure 1 shows the results of volumetric LA performance. Our results agree with those of the Literature^4^,^5^,^6^,^7^,^8^,^9^,^10^. Control and HCM volume curves are dramatically shifted being HCM volumes more than twofold dilated. Control mean volumetric trajectory shows a typical flattening before P–peak while it is absent in HCM. Global, reservoir and conduit ejection fractions are lowered in HCM, as illustrated by box plots. While booster pump ejection fraction seems not impaired, the absolute booster pump is increased in HCM. Controls and HCM differ for several 3DSTE parameters as shown in Supplementary Table 1 presented in Supplementary Online Appendix as already noted^4^, especially in some of the sub–regions automatically computed by Artida device. Our study was done, however, at homologous times and does not compare absolute minima or maxima as it might occur, in different individuals, at different times of their cycles. Figure 2 shows the necessity to adopt a PT procedure when performing shape analysis via classic GPA + PCA. It is evident in fact that, using classic GPA + PCA, PC scores explain concomitantly both inter- and intra- individual variation due to LA contraction. For example, at the positive extreme of PC1 we find a pathological individual whose cycle is limited to PC1 positive values, while other individual trajectories are all limited at the other PC1 extreme. When performing GPA in SSS + LS in SSS + PCA, as shown in Fig. 3, we clearly see what we expect from a cyclic deformation as heart revolution: a series of approximately elliptical trajectories, centered, in this case on the GM of the entire sample. Table 1 reports the variance explained by the first 10 PC scores. Supplementary Table 2 shows that, among the first 10 PC scores, individual significances slightly change during LA revolution. PC1 and PC2 (the dominant dimensions) are significant, again, in correspondence of 3rd and 4th homologous times.

The meaning of first 3 PC scores in terms of traditional 3DSTE parameters can be inferred from Supplementary Table 3 where univariate correlations between the first 3 PC scores and all 3DSTE parameters are reported. PC1 is, as expected, the most correlated PC with the majority of 3DSTE parameters, mainly with longitudinal and circumferential strains; however, PC2 and PC3 present correlations with torsional variables such as twist and rotation that, in at least some local atrial sub-regions, are significant under ANOVA (between Controls and HCM) at homologous times 3rd and 4th as shown in Supplementary Table 1. PC2 explains also a shear around vertical axis. Table 2 shows that in correspondence of ED times (3rd and 4th) Controls and HCM are significantly different under MANOVA.

**Figure 4:**
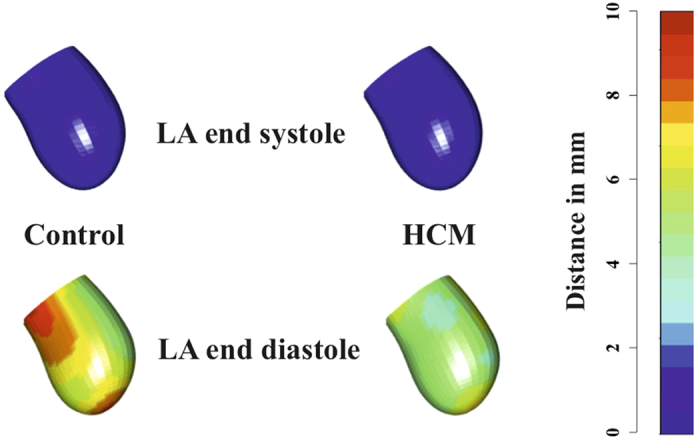
Differential LA deformation patterns in Controls and HCM. LA end-diastolic and end-systolic states for both Control and HCM subjects are shown. It is evident that in Controls we see a stronger contraction and deformation at end-diastole relative to HCM. For the sake of simplicity colormap refers, here, to the deformation relative to end-systolic state. Supplementary Video 1 shows the animation relative to the entire LA revolution.

Figure 4 shows the deformed states of Control and HCM LA in correspondence of 5th homologous time, thus at ED. The deformational difference is remarkable: Controls deform much more than HCM patients; this is evident not only on the purely homothetic component (i.e. the mere change in volume/size) but also in the rotational one (as also testified by 3DSTE ANOVAs in Supplementary Table 1) which is evident in the animation presented in Supplementary Video 1.

**Figure 5:**
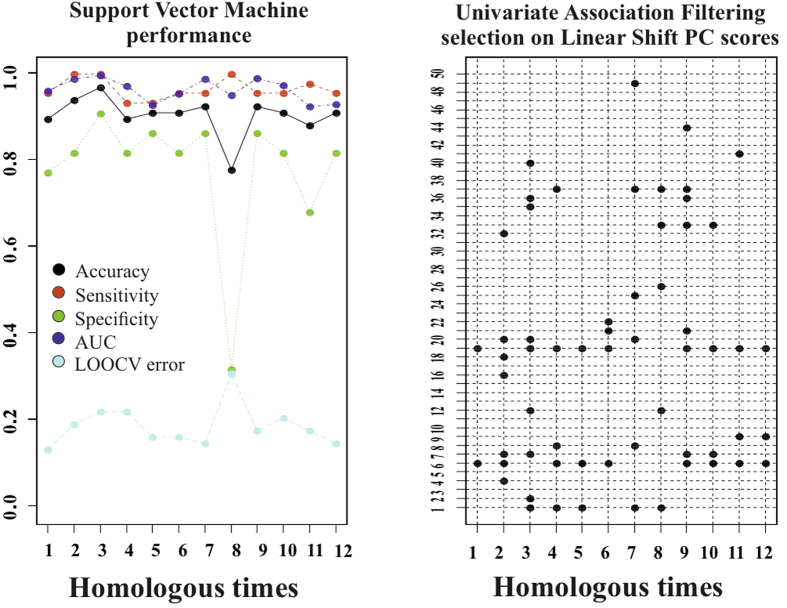
Classification performance of deformation analysis. The first 50 PC scores coming from the deformation analysis were subjected to UAF (see text) and then to SVM. Left: the specified performance measures of SVM in classifying Controls or HCM are shown at any homologous times. Right: PC scores retained at each SVM run, being found significant in UAF, at any homologous time.

**Figure 6:**
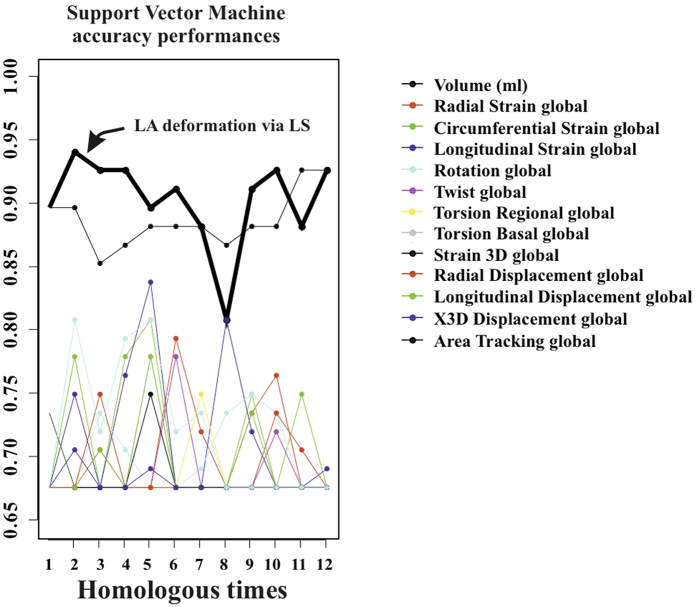
Comparison among accuracy performances. Accuracy of LA deformation via LS is manifestly above the accuracies of the other variables, even including volume, at most of the homologous times.

**Figure 7:**
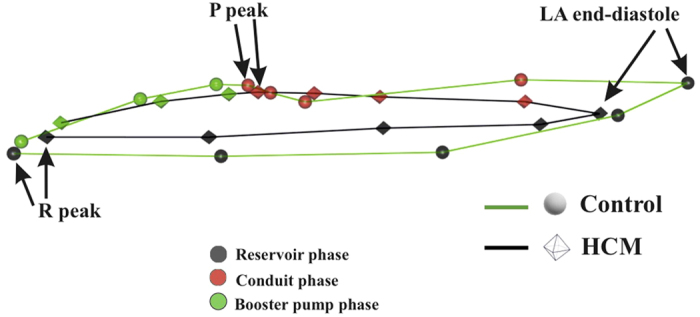
Actual mean trajectories shapes of Controls and HCM identified by the first 3 PC scores of LS analysis; it can be seen that both shape and size of the 2 trajectories are different. Strictly homologous times are indicated.

**Figure 8:**
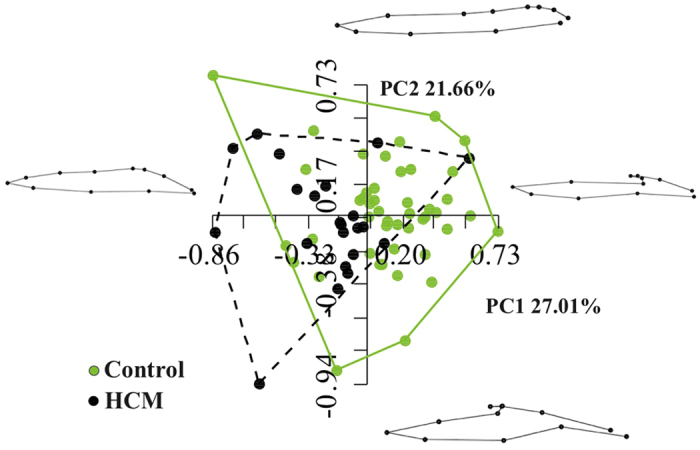
Shaping LA motion. PC1/PC2 space of GPA in SS + PCA on trajectory shapes is shown. Landmarks are the values of first 3 PC scores of the analysis shown in Fig. 3. Here SS is used as the pure trajectory shape is the objective of this analysis.

**Figure 9:**
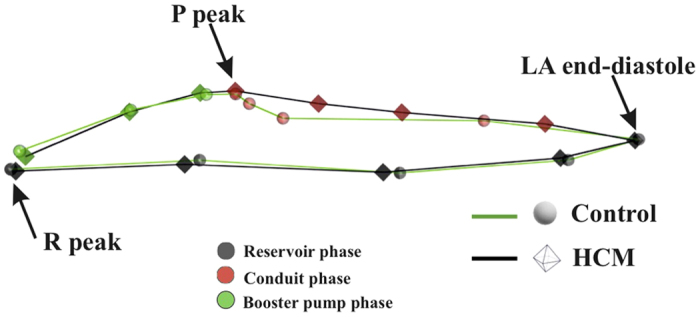
Mean trajectories explained by the PC1 (the sole significant under ANOVA).

**Figure 10:**
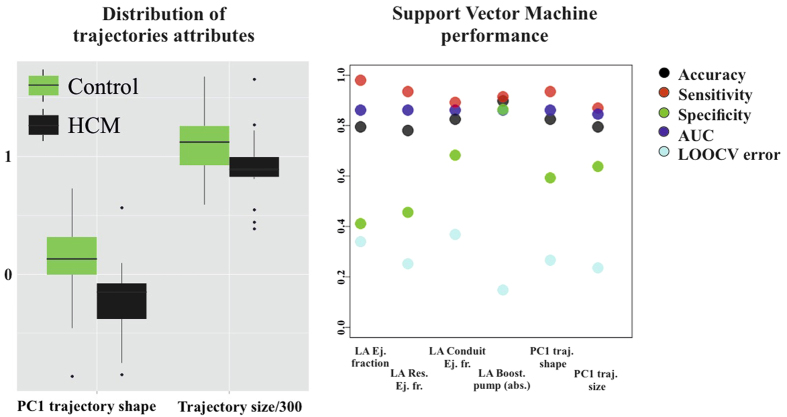
Classification performance of trajectories attributes. PC1 of trajectories shape analysis (the sole PC found significant in ANOVA) and trajectories size (significant in ANOVA) were subjected to SVM. No homologous times are present here as these attributes refer to the entire LA cycle and not to a particular time frame.

Classification performance of SVM applied to LS PC scores is shown in Fig. 5. Total accuracy reaches, at homologous time 3th about 97% with sensitivity always larger than specificity. LOOCV is almost constantly (except for homologous time 8th) at about 18%. AUC reaches 0.99 at homologous time 3rd. Figure 6 shows accuracy performance depicted in Fig. 5 when compared with the same analysis performed on classical 3DSTE global parameters. LS PC scores accuracy performs much better than global 3DSTE variables and than volume itself. The mean trajectories of Controls and HCM patients identified by the first 3 PC scores coming from GPA in SSS + LS in SSS + PCA (whose PC1/PC2 space was shown in Fig. 2) and treated as landmarks are shown in Fig. 7. The difference in both size and shape of the 2 trajectories can be easily seen. In fact, trajectory analysis shows that this difference is statistically significant. PC1/PC2 space of trajectory shape analysis is shown in Fig. 8. The sole PC1 is significant under individual ANOVA (*R*^2^: 0.21; p–value: 0.001); however, MANOVA using the first 10 PC scores (that explain, collectively about 90% of total variance) remains significant (Multivariate *R*^2^: 0.07; p–value: 0.002). Figure 9 shows that PC1 explains a specific trajectory morphology: the bending of the trajectory in correspondence of landmarks 6–9; these correspond to deformations occurring around the P peak that represents a crucial step in LA function (see discussion). PC1/PC2 and PC1/PC3 angles of trajectories orientation were not significant under ANOVA. We correlated trajectory’s shape PC1 and trajectory’s size with volumetric indicators. Results (Spearman) are shown in Table 3. Classification via SVM was applied to PC1 of trajectory shape analysis (the sole PC significant under ANOVA) and trajectory size (significant under ANOVA). SVM results are shown in Fig. 10. Accuracies of trajectory’s shape PC1 and size are 83% and 79% while AUCs are 0.86 and 0.84, respectively. These parameters have, on average, a better classification performance than the majority of volumetric indicators.

## Discussion

Shape and deformation analyses here presented were not only useful to characterise and discriminate HCM patients from Controls, but they also had a much higher accuracy and/or diagnostic performance than standard volumetric evaluation. While that LA volume and function are impaired in HCM patients is not a new notion^4^,^5^,^6^,^7^,^8^,^9^,^10^,^13^, shaping the motion via trajectory analysis provided, for the first time, parameters with a high classification power. Apart from classification, the greatest advantage is anyhow represented by the pathophysiological interpretation of LA functional role that our approach might carry. This could also have important translational consequences. The most intriguing evidence of the trajectory shape analysis is the position of the bending occurring before the P peak that is present in Controls and absent in HCM (Figs 7 and 9). This electrical event is of particular importance in healthy LA functioning: at this time we see the shift from the conduit LA function to the booster pump function^14^. This meso–diastolic transition is crucial during the active contractile chamber that augments LV ventricular filling in late diastole and acts a suction source that refills LA in early systole. The difference between Controls and HCM may indicate that the conduit function is impaired not only in terms of extent of LA volumetric performance; indeed, the way the contraction is achieved is expressed by the trajectory shape that, as stated above, is particularly different between the 6th an 9th homologous times measured starting from R–peak. Looking at Figs 7 and 9 enables to see that Control and HCM trajectories shapes begin to diverge at the interval between the 6th and 7th landmarks. Controls bend there downward so that between the 7th and 9th landmarks the trajectory rises. In fact, as shown in Fig. 2, the Conduit function is significantly impaired in HCM and it is the volumetric indicator most correlated with trajectory’s shape PC1. On the opposite, trajectory’s size, that is smaller in HCM, is correlated mostly with reservoir function, the second volumetric indicator that separates Controls from HCM.

It is important to note here that the shape of the trajectory as depicted in our Figs 7 and 9 was reconstructed in the present context by using the first 3 PCs after LS procedure. These explain collectively about 71% of total variance. However, the procedure we describe here can be effectively applied to more than 3 PCs. GPA on trajectory shapes can be performed on hyper-shapes identified by *n* dimensions, thus by using *n* PCs. A difficulty rises in this case as the hyper-shape cannot be illustrated and the interpretation of results requires a higher level of mathematical abstraction. This could limit the accessibility of this approach to a wide scientific community. It is for further investigations to look for classification performance of this more advanced approach.

These signals may be detected using LA volume, albeit LS deformation analysis better discriminates the pathology as shown in Fig. 6 and outperforms classic 3DSTE parameters. Indeed, these latter deliver information about LA deformation; however, 3DSTE deformation is evaluated at each time frame (in general, not a homologous time) by comparison with respect to the LA systolic state, a sort of blank that is 0 for all, by definition. As we centered shapes on GM, diastolic and systolic states are both recognized as deformed states and both are put in contrast with a real shape. Our entire procedure makes sense solely upon the identification and interpolation of homologous times^11^,^12^. Indeed, only temporal homology can guarantee the rationality of comparing deformed states. Interestingly, a cross correlation with LV deformational change might be done in order to explore the morphological and deformational covariance between LA and LV. It was recently shown^15^ that in HCM the transmitral flow velocity is much reduced in correspondence of P-peak, just the point where we observed the absence of bending in LA trajectory shape. We hypothesize, therefore, that the functional/morphological covariation between LA and LV express there its maximum potential to be further investigated in a conjoint analysis to derive potentially important translational information. In this way the hypothesis could be tested that a break in the natural deformational covariance between the 2 chambers is present in diseased conditions such as HCM and other diseases^14^,^15^,^16^.

## Methods

### Subjects and Ethic statements

The study was conducted after the approval of the “Dipartimento di Scienze Cardiovascolari, Respiratorie, Nefrologiche, Anestesiologiche e Geriatriche, Sapienza–Università di Roma” after submitting the protocol, as an observational spontaneous investigation, to the pertinent local Ethical Committee. All methods were carried out in accordance with the ethical guidelines of the Declaration of Helsinki. Written informed consent was obtained from each subject and 68 subjects were studied. For 46 healthy subjects we assessed, based on an accurate cardiological visit, the absence of any type of known heart disease. There were 22 non-obstructive HCM patients. Table 4 shows descriptive statistics of the sample under study.

### 3D data acquisition

The acquisition of LA geometry is achieved by means of 3DSTE, a technique that is today a consolidated standard in cardiological diagnosis, allowing the real time evaluation of LA and LV motion and the measurements of several diagnostic parameters as twist, torsion and longitudinal strain among others. LA geometrical data are collected during the cardiac revolution by means of PST–25SX Artida device, Toshiba Medical Systems Corp., Tokyo, Japan. LA geometries are reconstructed starting from a set of 6 homologous landmarks indentified on the endocardial layer, manually detected by the operator for all subjects under study. The same operator (AE) is involved in LA geometry reconstruction. Additional details and discussion about the comparison between fully automated or manual reconstruction can be found elsewhere^11^,^12^. The final dataset of any subject is a time-sequence of shapes, each composed by 1297 landmarks (assumed to be homologous) for endocardial surfaces, positioned along 36 horizontal circles, each composed of 36 landmarks, plus the apex. We get the landmark cloud (upon which the standard rotational, torsional and strain parameters are normally computed and outputted by any Artida device) due to an unlocked version of the software equipping our PST–25SX Artida, thanks to a special opportunity provided in the context of an official research and development agreement between the Dipartimento di Scienze Cardiovascolari, Respiratorie, Nefrologiche Anestesiologiche e Geriatriche, Sapienza Università di Roma and Toshiba Medical System Europe, Zoetermeer, The Netherland.

### Temporal homology and Interpolation

**Figure 11:**
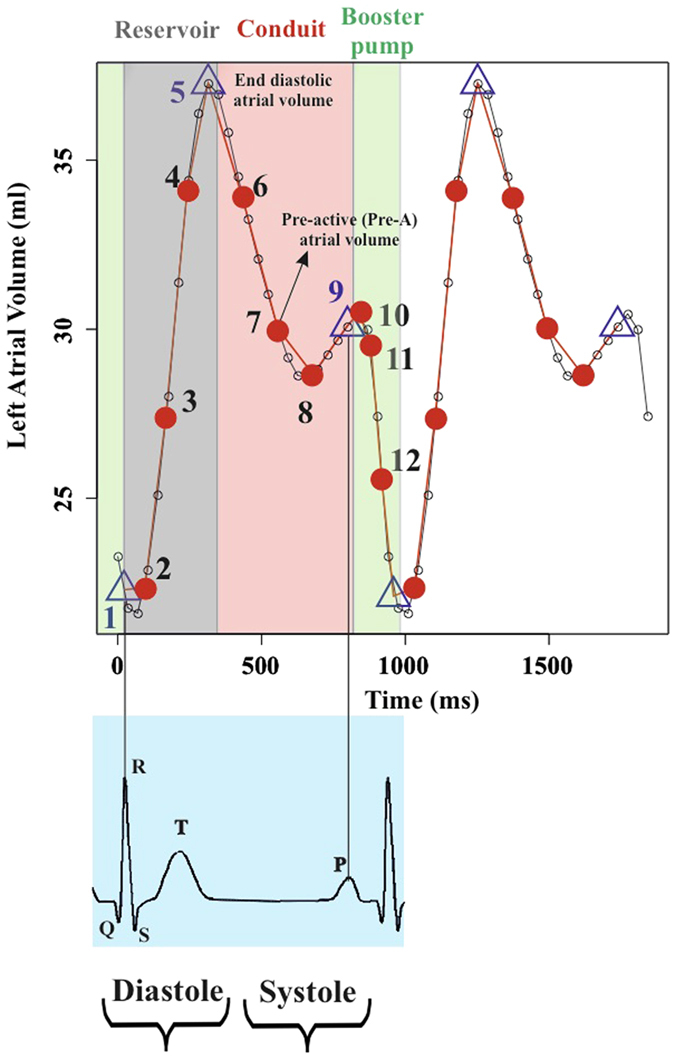
LA volume curve along 2 cardiac cycles. Small dots identify the times at which 3DSTE acquisition is done (observed times). Among these, the 3 strictly homologous times are identified by a triangle, whereas the others homologous times by a red dot. All in all, a fine reconstruction of the actual LA trajectory is obtained.

The first step of our method aims to introduce the concept of homologous times^11^,^12^. As we used Ecocardiographic data, we had the possibility to use the electrocardiogram associated to it in order to identify homologous times. The same cannot be applied when using MRI and peculiar spatiotemporal registration techniques are needed in order to standardize different individuals at comparable temporal intervals^17^,^18^. Among others, a spatio-temporal registration method was proposed, based on some specific physiological events^19^. Identifying and defining homologous times is a crucial step for correctly comparing any type of variable among different individuals acquired at different temporal resolutions and thus composed by different number of shapes in any cycle. Temporal homology fully extends in 4D the concept of anatomical homology and allows comparing not only shapes varying in time but also the shape of the trajectories themselves. Figure 11 shows the 12 homologous times chosen to interpolate LA univariate (3DSTE parameters) and multivariate (shapes) descriptors. Specifically, we have 3 strictly homologous times representing specific electromechanical events, *i.e.* R peak, LA end–diastolic volume, and P–peak; their exact time (in ms) were visually identified on each individual 3DSTE video clip. These 3 times are critical in LA functioning as they separate the 3 main LA functions occurring in the cardiac revolution, as Fig. 11 shows: reservoir (grey), conduit (pink) and active booster pump (green)^14^. The other times are obtained by sampling 3 equally spaced times between successive and strictly homologous times. As shown in Fig. 11, this approach guarantees to have a complete representation of LA trajectory during cardiac revolution. In order to reconstruct shapes, we performed separate Generalized Procrustes Analysis (GPA) in the Size and Shape Space (SSS), *i.e.* without scaling at unit size, followed by a Principal Component Analysis (PCA); then, we interpolated the full set of PC scores for each individual. GPA centers and optimally rotates shapes, optionally scaling to unit size, in order to remove non-shape attributes^20^. Typically, GPA is followed by PCA performed on aligned coordinates; PCA can be linear or non–linear and allows to get a ranking of the main shape–change modes and to visualize main shape–change directions. We used GPA in SSS rather than in the Shape Space (SS), thus without scaling shapes at unit size, as LA size is an important attribute of LA function. Individuals PC scores and PC loadings got from PCA are used to reconstruct LA shapes and sizes at any homologous time, thus obtaining a set of 12 shapes (each composed of 1297 landmarks) for each of the 68 subjects under study. The collection of these shapes is the object of the successive deformation analysis.

The SSS is less used than the SS but its geometrical structure is simpler. Full details can be found in elsewhere refs 20, 21, 22 apex. Here we can only summarize its geometrical genesis and some of its important features. We recall that the SS is built starting from the Euclidean space of *k* × *m* (*k* = number of landmarks, *m* = number of dimensions) configuration matrices via three operations that we can consider (without entering in more subtle mathematical distinctions), as projections: centering via translation, alignment via rotations, scaling to unit centroid size. While the first projection does not change the Euclidean structure of the space, the second and third make the SS a Non Euclidean Space. In particular, the third operation projects each configuration on an hyper-sphere of unit radius. The major consequence of this construction is that, after aligning two configurations, the geodesic line joining it, is an arch of circumference. In order to approximate the curved space around a given point (shape) one can project back each shape to the tangent space to the hyper–sphere on that point. The SSS can be built in the same way, but performing only the first two geometrical operations (translation and alignment) without projecting on the hyper–sphere. It is easy to understand that this procedure, leads, roughly speaking, to a *less Non Euclidean* space. In particular, in the SSS the geodesics joining two aligned configurations are straight lines: i.e. the same of the Euclidean configuration space. The consequence is that no projection is necessary toward the tangent space because a segment joining two aligned configuration coincides directly with a vector of the tangent space to the SSS on one of the two configurations. Practically this means that, once GPA is performed, the resulting configurations lie on the tangent space at the Grand Mean. This allows us to perform directly a PCA.

### Volumetric and deformation analysis

Albeit we do not focus on LA volume impairments, as done by previous investigators^4^,^5^,^6^,^7^,^8^,^9^,^10^, it is essential to extract LA volume indicators in order to correlate them with morphometric descriptors that we introduce and describe below. We thus computed the main LA functions using volumes corresponding to the various LA phases as described in Table 4, and showed them in Fig. 1; therein, the difference between absolute booster pump function and the corresponding ejection fraction can be appreciated. A crucial step in a deformation analysis is the management of inter–individual differences, which have to be eliminated; indeed, when different LA geometries moving in time are compared, classic GPA and PCA mix intra– and inter– individual variation, thus inhibiting the appreciation of pure deformation patterns. To this end, we follow a Linear Shift (LS) strategy^11^,^12^,^23^. LS consists in the application of the Parallel Transport (PT) tool in the Euclidean tangent plane to the Riemannian space of the shapes, instead of applying it in the Riemannian space itself^22^,^24^; it introduces an approximation which was however justified with specific reference to the echocardiographic data^11^,^12^.

LS strategy allows to estimate, hierarchically, the deformations occurring within each individual and to apply them to a *mannequin*. Specifically, we identify as our mannequin the Grand Mean (GM) of the entire sample, and use LS to apply individual deformation occurring within each cycle to the GM. It is worth noting that, as 3DSTE strain data are measured for each individual with respect to the end–systolic (ES) frame, typically 3DSTE *mannequin* is the ES shape and ES state is not a deformed state. Using GM as mannequin, instead of ES, allows to appreciate both ES and end-diastole (ED) as deformed states. Thus the sequence of deformation analysis consists of (GPA in SSS) + (LS in SSS) + PCA. Additional details about PT and LS can be found elsewhere^11^,^12^,^22^,^23^. The outcome of this procedure is a series of deformations of GM (as standard 3DSTE procedure delivers a series of deformations with respect to the ES state). Close points in the resulting PCA plot do not correspond to individuals that have the same shape but indicate individuals that are experiencing the same deformation.

### Trajectory analysis

As 3DSTE landmarks identify the shape of LA through a cloud of points, likewise PC scores values of LS analysis represent new landmarks which identify the shape (approximately elliptical) of each individual trajectory^11^,^12^,^25^,^26^. In this sense, we say that trajectory analysis aims at *shaping* LA motion. Thanks to the notion of homologous times and to the elimination of inter–individual differences via LS, we have a set of deformation trajectories, each composed of an equal number of individual shapes in correspondence of homologous electrophysiological times. We use the first 3 PC scores of LS analysis as landmarks which identify a 3D configuration for each trajectory, and study these latters by exploring their shapes, orientations and sizes. It is worth noting that these new landmarks are still homologous, due to the homologous time interpolation procedure described above.

We study trajectory shapes by performing a second–order GPA in SS + PCA on these shapes. During the GPA only landmarks 1, 5, 9, corresponding to the strictly homologous times identified in Fig. 11, were used to estimate optimal alignment, while the other homologous times are excluded during Procrustes distance minimization process, and are passively appended to GPA transformations (translation, scaling and rotation). This strategy allows to compare relatively complete shapes of trajectories without adding noise due to non perfectly homologous physiologically–based event estimation. PC scores deriving from such analysis are not referred to LA shape but to LA trajectory shape. Trajectory size can be evaluated by performing an ANOVA (between Controls and HCM) on the Centroid Size (CS) of trajectories’ configurations. Trajectory orientation is estimated by measuring the direction on PC1/PC2 and PC1/PC3 planes of the vectors identified by the first landmark, i.e. the PC scores values corresponding to R peak and the fifth landmark, i.e. PC scores values corresponding to LA ED. This strategy permits a full evaluation of the LA motion attributes.

### Linear models and classification

We used a series of ANOVAs on descriptive 3DSTE parameters in order to assess differences between Controls and HCM at any homologous time. The same was done for the first 10 PC scores of deformation analysis; on these we also performed the multivariate test via MANOVAs.

The first 3 PC scores of deformation analysis were also correlated with per–individual mean centered 3DSTE variables in order to assess their significance in terms of traditional 3DSTE parameters. ANOVAs were also performed on PC1/PC2 and PC1/PC3 angles and on trajectory size, while MANOVA was performed on the first 5 PC scores of trajectory shape (albeit reporting them also for univariate significances). Deformation PC scores (at any homologous time) and trajectories’ attributes were also subjected to classification analysis in order to assess the potential of these indicators in recognizing HCM condition. As shown previously^27^, Support Vector Machine (SVM) outperforms classic classification procedures (logistic regression, discriminant analysis and random forest, among others) in discriminating healthy LV from infarcted patients using data very similar to those used here. For this reason, we used SVM with radial basis Gaussian kernel function, hyperparameter *C* = 1, and with reference to the first 50 LS PC scores evaluated at any homologous time, in order to classify our sample. At any time a Univariate Association Filtering (UAF) with p-value cut-off at 0.05 (thus pretty conservative in a classification problem) was performed as feature selection. The same was done for trajectory attributes. In the trajectory attributes’ analysis there are no homologous times as they are not related to any single homologous time, being representative of the entire LA revolution. Any SVM run was evaluated in terms of total accuracy, sensitivity, specificity, Area Under the Curve (AUC) and Leave One Out Cross Validation error (LOOCV).

## Conclusions

This study sought to test the hypotheses that LA deformation path is impaired in HCM, and that recognizable abnormalities are present not only in deformation but also in motion trajectory. Indeed, whereas recent studies of LA dysfunction in HCM suggest that LA dilation is augmented while LA strains are reduced^4^,^5^,^6^,^7^,^8^,^9^,^10^,^13^, no investigations were performed on full LA deformation and whole LA deformational trajectory in time.

We enrolled 22 HCM patients and 46 Healthy subjects. We used 3D Speckle Tracking Echocardiography (3DSTE) derived landmark clouds in order to perform full shape and deformation analyses. This was achieved using Geometric Morphometrics and Parallel Transport that allow to appreciate solely the deformation without the noise due to inter–individual differences^11^,^12^. We also used the notion of temporal homology in order to interpolate shapes at physiologically comparable times for each individual. Thus not only deformation per se but also the shape of the deformation trajectories during LA revolution were assessed. Both deformation analysis scores and trajectory shape analysis scores underwent classification via SVM in order to test the discriminating power of these parameters in characterizing HCM.

We showed that deformation analysis was highly effective in recognizing HCM: AUC during meso-diastole reached 0.99 and total accuracy 0.81. Trajectory shape and trajectory size were much different in Controls versus HCM and their classification power was high in terms of AUC (0.87 and 0.81 respectively). The two trajectories were particularly different just before the transition between LA conduit and booster pump functions. So, full shape and deformation analyses accompanied by a trajectory analysis allow a straightforward perception of pathophysiological consequences of HCM condition.

It is provocative that by shape analysis traslational differences might be appreciated of a peculiar pathophysiological time frame in LA function that we showed abnormal in HCM, confirming results obtained by different methods^15^, but whose approach might be extended to different conditions such as congestive heart failure whereby LA function might so be disclosed as a potentially important target for individualised therapeutical interventions^16^. It might be worthwhile to apply the complex techniques that we described to look for novel pathophysiological approaches in order to better define atrio–ventricular interaction.

## Additional Information

**How to cite this article**: Piras, P. *et al*. Left Atrial trajectory impairment in Hypertrophic Cardiomyopathy disclosed by Geometric Morphometrics and Parallel Transport. *Sci. Rep.* **6**, 34906; doi: 10.1038/srep34906 (2016).

## Supplementary Material

Supplementary Information

Supplementary Video

## Figures and Tables

**Table 1:** Variance explained by first 10 PC scores coming from GPA in SSS + LS + PCA.

PCscores	Eigenvalues%	Variance%	Cumulative%
PC1	7667.726	62.504	62.504
PC2	612.476	4.993	67.496
PC3	469.388	3.826	71.322
PC4	404.313	3.296	74.618
PC5	304.468	2.482	77.100
PC6	288.151	2.349	79.449
PC7	265.016	2.160	81.609
PC8	230.903	1.882	83.491
PC9	162.446	1.324	84.816
PC10	155.662	1.269	86.085

**Table 2:** MANOVAs (using the first 10 PC scores) on PC sores coming from GPA in SSS + LS + PCA evaluated at each homologous time.

Homologous times	R–sq	P–value
*t*_1_	0.036	**0.026**
*t*_2_	0.030	0.054
*t*_3_	0.051	**0.007**
*t*_4_	0.049	**0.002**
*t*_5_	0.060	**0.001**
*t*_6_	0.027	0.078
*t*_7_	0.071	**0.010**
*t*_8_	0.033	0.075
*t*_9_	0.016	0.331
*t*_10_	0.019	0.238
*t*_11_	0.026	0.114
*t*_12_	0.041	**0.014**

**Table 3:** Spearman correlations between trajectory’s size and PC1 of trajectory shape analysis with volumetric indicators.

Volumetric indicators	Trajectory size	p–val	PC1 trajectory shape	p–val
Global ejection fraction	0.796	0.001	0.319	0.008
Reservoir ejection fraction	0.813	0.001	0.29	0.017
Conduit ejection fraction	0.515	0.001	0.855	0.001
Booster pump ejection fraction	0.386	0.001	−0.604	0.001
Booster pump absolute	−0.489	0.001	−0.448	0.001

**Table 4:** Descriptive statistics ± standard deviation for the populations under study.

Descriptive parameter	Controls = 46	HCM = 22
Age (%)	39 ± 8.33	48 ± 12.5
EF (%)	55.7 ± 0.07	43.5 ± 0.13
Interventricular septum (mm)	8.47 ± 1.43	18.13 ± 3.99
Sex (males/females)	30/16	14/8
Beat rate (beats/min)	77 ± 13.16	76 ± 13.33
Body surface area (mm^2^)	1.86 ± 0.20	1.85 ± 0.18
**Genetic mutations for HCM**		
MYBPC3	—	7
MYH7	—	4
TNNT2	—	3
Mutation not known	—	6
Not investigated	—	2
