# Supplementary material for: Left Atrial trajectory impairment in Hypertrophic Cardiomyopathy disclosed by Geometric Morphometrics and Parallel Transport
Source: Sci Rep. 2016 Oct 7;6:34906. doi: 10.1038/srep34906 (PMC5054674; doi:10.1038/srep34906)
Supplement: Supplementary Information [file srep34906-s1.doc]

| **Supplementary Table 1. Univariate ANOVAs between Control and HCM for each homologous time for all 3DSTE parameters. Two blocks each with 6 homologous times are showed in sequence. For any time *p*-values, significance flag -*- and the difference between Control and HCM are shown.** | | | | | | | | | | | | | | | | | | |
| --- | --- | --- | --- | --- | --- | --- | --- | --- | --- | --- | --- | --- | --- | --- | --- | --- | --- | --- |
| **VARIABLES** | **P.val1** | **Sig1** | **Contr-HCM1** | **P-val2** | **Sig2** | **Contr-HCM2** | **P-val3** | **Sig3** | **Contr-HCM3** | **P-val4** | **Sig4** | **Contr-HCM4** | **P-val5** | **Sig5** | **Contr-HCM5** | **P-val6** | **Sig6** | **Contr-HCM6** |
| Volume.mL. | 0.000 | * | -39.065 | 0.000 | * | -41.421 | 0.000 | * | -44.978 | 0.000 | * | -48.130 | 0.000 | * | -49.122 | 0.000 | * | -51.163 |
| Myo.Vol_.mL. | 0.000 | * | -20.884 | 0.000 | * | -21.551 | 0.000 | * | -23.857 | 0.000 | * | -24.361 | 0.000 | * | -24.442 | 0.000 | * | -25.299 |
| RStrain_BA | 0.868 |  | -0.159 | 0.852 |  | 0.515 | 0.978 |  | -0.122 | 0.682 |  | 1.811 | 0.731 |  | 1.318 | 0.804 |  | 0.782 |
| RStrain_BAS | 0.654 |  | 0.304 | 0.221 |  | 2.868 | 0.611 |  | 2.085 | 0.703 |  | 1.903 | 0.810 |  | 1.040 | 0.660 |  | 1.459 |
| RStrain_BS | 0.633 |  | 0.299 | 0.858 |  | 0.367 | 0.405 |  | -3.668 | 0.392 |  | -4.080 | 0.115 |  | -6.598 | 0.082 |  | -5.890 |
| RStrain_BI | 0.939 |  | 0.045 | 0.912 |  | -0.256 | 0.455 |  | -3.367 | 0.741 |  | -1.582 | 0.558 |  | -2.499 | 0.216 |  | -3.698 |
| RStrain_BP | 0.978 |  | 0.022 | 0.082 |  | -4.566 | 0.030 | * | -8.778 | 0.135 |  | -6.418 | 0.176 |  | -5.543 | 0.064 |  | -5.938 |
| RStrain_BL | 0.872 |  | -0.137 | 0.232 |  | -4.403 | 0.049 | * | -8.825 | 0.029 | * | -8.658 | 0.041 | * | -8.281 | 0.046 | * | -7.316 |
| RStrain_MA | 0.961 |  | -0.037 | 0.683 |  | -1.019 | 0.381 |  | -3.030 | 0.682 |  | 1.589 | 0.902 |  | 0.442 | 0.671 |  | 1.325 |
| RStrain_MAS | 0.606 |  | 0.304 | 0.710 |  | -1.116 | 0.026 | * | -11.108 | 0.087 |  | -9.906 | 0.210 |  | -6.610 | 0.139 |  | -6.600 |
| RStrain_MS | 0.163 |  | 0.909 | 0.312 |  | 2.902 | 0.925 |  | 0.456 | 0.801 |  | 1.254 | 0.984 |  | 0.091 | 0.472 |  | -2.644 |
| RStrain_MI | 0.703 |  | 0.257 | 0.076 |  | 3.749 | 0.015 | * | 7.494 | 0.012 | * | 10.131 | 0.056 |  | 7.317 | 0.109 |  | 4.245 |
| RStrain_MP | 0.494 |  | -0.437 | 0.064 |  | -3.958 | 0.120 |  | -4.949 | 0.850 |  | -0.832 | 0.787 |  | -1.093 | 0.764 |  | -1.018 |
| RStrain_ML | 0.894 |  | -0.095 | 0.101 |  | -4.899 | 0.007 | * | -10.157 | 0.056 |  | -7.701 | 0.068 |  | -7.271 | 0.251 |  | -4.565 |
| RStrain_AA | 0.575 |  | 0.499 | 0.789 |  | -0.865 | 0.221 |  | -7.082 | 0.644 |  | -3.617 | 0.998 |  | -0.026 | 0.924 |  | -0.632 |
| RStrain_AS | 0.483 |  | 0.622 | 0.490 |  | 2.228 | 0.971 |  | -0.272 | 0.741 |  | 4.531 | 0.625 |  | 9.737 | 0.704 |  | 4.941 |
| RStrain_AI | 0.968 |  | 0.028 | 0.798 |  | -0.484 | 0.588 |  | -2.249 | 0.851 |  | 0.881 | 0.851 |  | 0.822 | 0.952 |  | 0.229 |
| RStrain_AL | 0.932 |  | -0.074 | 0.440 |  | -2.130 | 0.081 |  | -8.423 | 0.170 |  | -6.316 | 0.212 |  | -5.706 | 0.236 |  | -4.815 |
| RStrain_global | 0.736 |  | 0.147 | 0.610 |  | -0.692 | 0.077 |  | -3.875 | 0.527 |  | -1.686 | 0.610 |  | -1.429 | 0.378 |  | -1.883 |
| CStrain_BA | 0.282 |  | 0.865 | 0.009 | * | 6.449 | 0.002 | * | 13.831 | 0.001 | * | 19.927 | 0.001 | * | 21.297 | 0.006 | * | 13.181 |
| CStrain_BAS | 0.273 |  | 1.133 | 0.035 | * | 6.045 | 0.015 | * | 12.166 | 0.003 | * | 19.103 | 0.003 | * | 19.672 | 0.035 | * | 11.404 |
| CStrain_BS | 0.233 |  | 1.257 | 0.459 |  | 2.008 | 0.802 |  | 0.871 | 0.329 |  | 3.795 | 0.061 |  | 7.845 | 0.089 |  | 6.376 |
| CStrain_BI | 0.445 |  | 0.655 | 0.690 |  | -0.812 | 0.151 |  | -4.874 | 0.089 |  | -7.421 | 0.086 |  | -7.602 | 0.062 |  | -7.335 |
| CStrain_BP | 0.343 |  | 1.170 | 0.049 | * | 5.670 | 0.048 | * | 9.082 | 0.124 |  | 9.112 | 0.110 |  | 9.827 | 0.677 |  | 2.304 |
| CStrain_BL | 0.096 |  | 1.402 | 0.001 | * | 9.432 | 0.000 | * | 19.387 | 0.000 | * | 28.254 | 0.000 | * | 33.643 | 0.000 | * | 22.607 |
| CStrain_MA | 0.666 |  | 0.326 | 0.155 |  | 3.027 | 0.061 |  | 6.293 | 0.014 | * | 12.149 | 0.006 | * | 15.728 | 0.021 | * | 9.271 |
| CStrain_MAS | 0.147 |  | 1.551 | 0.078 |  | 4.414 | 0.037 | * | 8.380 | 0.004 | * | 14.262 | 0.001 | * | 16.931 | 0.048 | * | 8.071 |
| CStrain_MS | 0.186 |  | 0.932 | 0.300 |  | 2.445 | 0.714 |  | 1.288 | 0.589 |  | 2.203 | 0.304 |  | 4.141 | 0.972 |  | 0.121 |
| CStrain_MI | 0.460 |  | 0.441 | 0.391 |  | 1.371 | 0.940 |  | -0.201 | 0.336 |  | -3.465 | 0.532 |  | -2.426 | 0.243 |  | -3.997 |
| CStrain_MP | 0.602 |  | 0.599 | 0.137 |  | 3.491 | 0.345 |  | 3.688 | 0.559 |  | 2.951 | 0.217 |  | 6.528 | 0.759 |  | 1.307 |
| CStrain_ML | 0.869 |  | 0.155 | 0.100 |  | 4.351 | 0.018 | * | 10.520 | 0.003 | * | 17.262 | 0.000 | * | 22.438 | 0.004 | * | 12.867 |
| CStrain_AA | 0.524 |  | 0.907 | 0.563 |  | 2.242 | 0.593 |  | 2.685 | 0.170 |  | 11.300 | 0.081 |  | 16.859 | 0.363 |  | 6.375 |
| CStrain_AS | 0.213 |  | 1.068 | 0.218 |  | 3.426 | 0.690 |  | 1.603 | 0.426 |  | 4.987 | 0.207 |  | 8.977 | 0.869 |  | 0.828 |
| CStrain_AI | 0.657 |  | 0.470 | 0.124 |  | 5.070 | 0.133 |  | 6.953 | 0.214 |  | 7.783 | 0.191 |  | 8.888 | 0.863 |  | 0.995 |
| CStrain_AL | 0.504 |  | 0.956 | 0.715 |  | 1.561 | 0.652 |  | 2.900 | 0.254 |  | 9.859 | 0.132 |  | 14.644 | 0.508 |  | 5.182 |
| CStrain_global | 0.282 |  | 0.867 | 0.026 | * | 3.763 | 0.009 | * | 5.910 | 0.001 | * | 9.503 | 0.000 | * | 12.336 | 0.039 | * | 5.597 |
| LStrain_BA | 0.578 |  | -0.222 | 0.015 | * | 4.070 | 0.001 | * | 9.039 | 0.004 | * | 9.963 | 0.022 | * | 8.106 | 0.040 | * | 5.512 |
| LStrain_BAS | 0.485 |  | -0.297 | 0.173 |  | 2.055 | 0.017 | * | 5.776 | 0.035 | * | 6.611 | 0.061 |  | 5.964 | 0.271 |  | 2.697 |
| LStrain_BS | 0.431 |  | 0.462 | 0.515 |  | 1.210 | 0.309 |  | 2.980 | 0.194 |  | 4.612 | 0.190 |  | 4.838 | 0.497 |  | 2.107 |
| LStrain_BI | 0.181 |  | 0.829 | 0.196 |  | 2.367 | 0.268 |  | 3.492 | 0.169 |  | 5.249 | 0.163 |  | 5.292 | 0.558 |  | 1.898 |
| LStrain_BP | 0.088 |  | 1.013 | 0.069 |  | 3.284 | 0.028 | * | 6.919 | 0.015 | * | 10.044 | 0.011 | * | 10.803 | 0.132 |  | 5.168 |
| LStrain_BL | 0.666 |  | 0.228 | 0.006 | * | 4.700 | 0.000 | * | 10.517 | 0.000 | * | 14.087 | 0.001 | * | 13.322 | 0.004 | * | 9.897 |
| LStrain_MA | 0.629 |  | -0.277 | 0.874 |  | 0.279 | 0.073 |  | 5.375 | 0.082 |  | 6.990 | 0.094 |  | 6.886 | 0.430 |  | 2.618 |
| LStrain_MAS | 0.136 |  | 0.944 | 0.055 |  | 3.467 | 0.004 | * | 9.258 | 0.006 | * | 11.961 | 0.008 | * | 11.962 | 0.079 |  | 6.073 |
| LStrain_MS | 0.056 |  | 1.465 | 0.014 | * | 4.781 | 0.051 |  | 6.761 | 0.145 |  | 6.322 | 0.090 |  | 7.227 | 0.610 |  | 1.702 |
| LStrain_MI | 0.171 |  | 1.106 | 0.022 | * | 4.850 | 0.058 |  | 7.108 | 0.365 |  | 4.048 | 0.328 |  | 4.316 | 0.873 |  | 0.549 |
| LStrain_MP | 0.588 |  | 0.391 | 0.083 |  | 3.351 | 0.102 |  | 5.520 | 0.218 |  | 4.816 | 0.102 |  | 6.307 | 0.336 |  | 2.918 |
| LStrain_ML | 0.459 |  | -0.434 | 0.800 |  | 0.412 | 0.218 |  | 3.687 | 0.168 |  | 5.261 | 0.124 |  | 5.885 | 0.595 |  | 1.672 |
| LStrain_AA | 0.776 |  | 0.278 | 0.051 |  | 5.104 | 0.021 | * | 8.812 | 0.027 | * | 12.385 | 0.009 | * | 16.244 | 0.039 | * | 9.721 |
| LStrain_AS | 0.429 |  | 0.459 | 0.169 |  | 3.049 | 0.305 |  | 3.464 | 0.425 |  | 3.847 | 0.408 |  | 4.298 | 0.856 |  | -0.590 |
| LStrain_AI | 0.992 |  | 0.005 | 0.306 |  | 2.692 | 0.171 |  | 5.699 | 0.266 |  | 5.736 | 0.204 |  | 6.411 | 0.581 |  | 2.173 |
| LStrain_AL | 0.952 |  | 0.055 | 0.303 |  | 3.021 | 0.136 |  | 7.002 | 0.112 |  | 9.696 | 0.030 | * | 14.138 | 0.093 |  | 9.847 |
| LStrain_global | 0.465 |  | 0.375 | 0.020 | * | 3.045 | 0.002 | * | 6.338 | 0.002 | * | 7.603 | 0.001 | * | 8.251 | 0.034 | * | 3.998 |
| Rotation_BA | 0.460 |  | -0.159 | 0.426 |  | -0.635 | 0.482 |  | -0.945 | 0.561 |  | -1.005 | 0.994 |  | 0.013 | 0.890 |  | -0.203 |
| Rotation_BAS | 0.216 |  | -0.326 | 0.079 |  | -1.509 | 0.322 |  | -1.419 | 0.357 |  | -1.588 | 0.321 |  | -1.659 | 0.112 |  | -2.153 |
| Rotation_BS | 0.625 |  | -0.129 | 0.840 |  | 0.151 | 0.356 |  | 1.070 | 0.119 |  | 2.037 | 0.044 | * | 2.539 | 0.397 |  | 1.004 |
| Rotation_BI | 0.337 |  | -0.283 | 0.011 | * | -2.633 | 0.003 | * | -4.250 | 0.003 | * | -4.256 | 0.014 | * | -3.394 | 0.027 | * | -2.514 |
| Rotation_BP | 0.180 |  | -0.362 | 0.003 | * | -3.037 | 0.000 | * | -5.573 | 0.000 | * | -7.006 | 0.000 | * | -6.948 | 0.000 | * | -5.664 |
| Rotation_BL | 0.266 |  | -0.257 | 0.037 | * | -1.750 | 0.026 | * | -3.139 | 0.009 | * | -4.296 | 0.012 | * | -4.104 | 0.005 | * | -4.217 |
| Rotation_MA | 0.044 | * | -0.489 | 0.105 |  | -1.223 | 0.405 |  | -1.007 | 0.526 |  | -1.001 | 0.623 |  | -0.801 | 0.405 |  | -1.126 |
| Rotation_MAS | 0.046 | * | -0.402 | 0.030 | * | -1.652 | 0.113 |  | -1.825 | 0.375 |  | -1.358 | 0.397 |  | -1.279 | 0.142 |  | -1.738 |
| Rotation_MS | 0.330 |  | -0.186 | 0.311 |  | -0.673 | 0.799 |  | -0.262 | 0.535 |  | 0.732 | 0.567 |  | 0.663 | 0.638 |  | -0.496 |
| Rotation_MI | 0.428 |  | -0.247 | 0.025 | * | -1.938 | 0.017 | * | -2.691 | 0.029 | * | -2.691 | 0.027 | * | -2.959 | 0.008 | * | -2.987 |
| Rotation_MP | 0.493 |  | -0.206 | 0.032 | * | -1.773 | 0.003 | * | -3.496 | 0.000 | * | -4.937 | 0.000 | * | -5.348 | 0.000 | * | -4.665 |
| Rotation_ML | 0.068 |  | -0.364 | 0.034 | * | -1.418 | 0.026 | * | -2.256 | 0.006 | * | -3.289 | 0.004 | * | -3.490 | 0.001 | * | -3.617 |
| Rotation_AA | 0.288 |  | -0.510 | 0.049 | * | -2.563 | 0.033 | * | -4.279 | 0.105 |  | -3.849 | 0.179 |  | -3.059 | 0.084 |  | -3.054 |
| Rotation_AS | 0.502 |  | -0.277 | 0.100 |  | -2.254 | 0.008 | * | -5.063 | 0.056 |  | -4.384 | 0.167 |  | -3.061 | 0.066 |  | -2.993 |
| Rotation_AI | 0.318 |  | -0.543 | 0.125 |  | -2.160 | 0.021 | * | -4.672 | 0.044 | * | -4.865 | 0.068 |  | -4.234 | 0.021 | * | -4.223 |
| Rotation_AL | 0.406 |  | -0.421 | 0.096 |  | -2.157 | 0.014 | * | -4.559 | 0.012 | * | -5.401 | 0.019 | * | -4.901 | 0.007 | * | -4.495 |
| Rotation_global | 0.122 |  | -0.323 | 0.006 | * | -1.701 | 0.001 | * | -2.771 | 0.004 | * | -2.947 | 0.012 | * | -2.627 | 0.002 | * | -2.695 |
| Twist_BA | 0.116 |  | -0.165 | 0.596 |  | 0.209 | 0.109 |  | 0.927 | 0.122 |  | 1.116 | 0.228 |  | 0.881 | 0.093 |  | 0.982 |
| Twist_BAS | 0.769 |  | -0.023 | 0.601 |  | 0.204 | 0.271 |  | 0.764 | 0.189 |  | 1.181 | 0.203 |  | 1.127 | 0.062 |  | 1.333 |
| Twist_BS | 0.523 |  | -0.063 | 0.981 |  | -0.008 | 0.101 |  | 0.947 | 0.125 |  | 1.077 | 0.298 |  | 0.687 | 0.220 |  | 0.697 |
| Twist_BI | 0.886 |  | -0.016 | 0.985 |  | -0.007 | 0.416 |  | 0.488 | 0.607 |  | 0.359 | 0.730 |  | -0.223 | 0.483 |  | -0.354 |
| Twist_BP | 0.243 |  | 0.106 | 0.818 |  | -0.085 | 0.680 |  | 0.270 | 0.948 |  | 0.049 | 0.620 |  | -0.346 | 0.313 |  | -0.540 |
| Twist_BL | 0.560 |  | 0.052 | 0.163 |  | 0.409 | 0.015 | * | 0.990 | 0.016 | * | 1.162 | 0.013 | * | 1.107 | 0.006 | * | 1.048 |
| Twist_MA | 0.096 |  | -0.477 | 0.760 |  | -0.293 | 0.534 |  | 0.927 | 0.461 |  | 1.229 | 0.884 |  | 0.239 | 0.870 |  | 0.227 |
| Twist_MAS | 0.606 |  | -0.113 | 0.993 |  | -0.008 | 0.843 |  | 0.312 | 0.494 |  | 1.320 | 0.481 |  | 1.341 | 0.269 |  | 1.597 |
| Twist_MS | 0.710 |  | -0.108 | 0.428 |  | -0.720 | 0.885 |  | -0.204 | 0.996 |  | -0.008 | 0.551 |  | -0.953 | 0.615 |  | -0.641 |
| Twist_MI | 0.954 |  | 0.021 | 0.565 |  | 0.654 | 0.218 |  | 1.946 | 0.273 |  | 1.798 | 0.924 |  | 0.140 | 0.517 |  | -0.821 |
| Twist_MP | 0.464 |  | 0.245 | 0.221 |  | 1.128 | 0.122 |  | 2.287 | 0.236 |  | 2.065 | 0.470 |  | 1.187 | 0.742 |  | 0.406 |
| Twist_ML | 0.840 |  | -0.049 | 0.332 |  | 0.728 | 0.100 |  | 1.878 | 0.081 |  | 2.160 | 0.147 |  | 1.681 | 0.146 |  | 1.578 |
| Twist_AA | 0.454 |  | -0.391 | 0.436 |  | -1.172 | 0.368 |  | -2.089 | 0.638 |  | -1.249 | 0.621 |  | -1.246 | 0.652 |  | -0.890 |
| Twist_AS | 0.749 |  | -0.166 | 0.203 |  | -2.027 | 0.036 | * | -4.617 | 0.094 |  | -4.422 | 0.140 |  | -3.750 | 0.214 |  | -2.365 |
| Twist_AI | 0.775 |  | -0.180 | 0.667 |  | 0.697 | 0.773 |  | 0.670 | 0.768 |  | 0.791 | 0.909 |  | 0.272 | 0.591 |  | -1.055 |
| Twist_AL | 0.889 |  | -0.085 | 0.965 |  | 0.058 | 0.907 |  | -0.227 | 0.891 |  | 0.304 | 0.792 |  | 0.566 | 0.696 |  | 0.718 |
| Twist_global | 0.708 |  | -0.088 | 0.983 |  | -0.013 | 0.720 |  | 0.330 | 0.599 |  | 0.558 | 0.866 |  | 0.169 | 0.882 |  | 0.121 |
| Torsion_Regional_BA | 0.081 |  | -0.227 | 0.972 |  | 0.017 | 0.289 |  | 0.703 | 0.339 |  | 0.751 | 0.630 |  | 0.383 | 0.515 |  | 0.433 |
| Torsion_Regional_BAS | 0.552 |  | -0.050 | 0.906 |  | 0.052 | 0.516 |  | 0.471 | 0.373 |  | 0.783 | 0.379 |  | 0.752 | 0.241 |  | 0.799 |
| Torsion_Regional_BS | 0.785 |  | -0.029 | 0.952 |  | -0.021 | 0.213 |  | 0.689 | 0.212 |  | 0.786 | 0.471 |  | 0.427 | 0.484 |  | 0.356 |
| Torsion_Regional_BI | 0.777 |  | 0.045 | 0.708 |  | 0.165 | 0.277 |  | 0.683 | 0.385 |  | 0.571 | 0.931 |  | -0.055 | 0.643 |  | -0.239 |
| Torsion_Regional_BP | 0.358 |  | 0.114 | 0.645 |  | 0.188 | 0.309 |  | 0.639 | 0.436 |  | 0.516 | 0.819 |  | 0.150 | 0.959 |  | -0.027 |
| Torsion_Regional_BL | 0.941 |  | -0.008 | 0.158 |  | 0.473 | 0.018 | * | 1.124 | 0.026 | * | 1.249 | 0.024 | * | 1.141 | 0.025 | * | 1.023 |
| Torsion_Regional_MA | 0.840 |  | -0.034 | 0.268 |  | -0.489 | 0.348 |  | -0.598 | 0.522 |  | -0.476 | 0.368 |  | -0.644 | 0.276 |  | -0.689 |
| Torsion_Regional_MAS | 0.740 |  | -0.047 | 0.478 |  | -0.306 | 0.128 |  | -0.892 | 0.336 |  | -0.562 | 0.553 |  | -0.338 | 0.552 |  | -0.276 |
| Torsion_Regional_MS | 0.964 |  | 0.006 | 0.071 |  | -0.738 | 0.002 | * | -1.799 | 0.006 | * | -1.704 | 0.020 | * | -1.371 | 0.045 | * | -1.025 |
| Torsion_Regional_MI | 0.939 |  | -0.013 | 0.375 |  | 0.375 | 0.605 |  | 0.285 | 0.536 |  | 0.385 | 0.495 |  | 0.371 | 0.888 |  | 0.070 |
| Torsion_Regional_MP | 0.953 |  | 0.014 | 0.080 |  | 0.881 | 0.207 |  | 0.867 | 0.298 |  | 0.759 | 0.314 |  | 0.691 | 0.305 |  | 0.613 |
| Torsion_Regional_ML | 0.857 |  | -0.034 | 0.567 |  | -0.239 | 0.416 |  | -0.555 | 0.509 |  | -0.532 | 0.429 |  | -0.586 | 0.520 |  | -0.392 |
| Torsion_Regional_AA | 0.296 |  | 0.269 | 0.994 |  | -0.006 | 0.276 |  | -0.928 | 0.193 |  | -1.075 | 0.296 |  | -0.705 | 0.332 |  | -0.536 |
| Torsion_Regional_AS | 0.582 |  | 0.102 | 0.919 |  | 0.053 | 0.394 |  | -0.730 | 0.260 |  | -1.090 | 0.409 |  | -0.677 | 0.687 |  | -0.295 |
| Torsion_Regional_AI | 0.965 |  | 0.007 | 0.705 |  | -0.221 | 0.277 |  | -0.763 | 0.396 |  | -0.649 | 0.817 |  | 0.153 | 0.435 |  | 0.493 |
| Torsion_Regional_AL | 0.613 |  | 0.103 | 0.641 |  | -0.401 | 0.292 |  | -0.878 | 0.547 |  | -0.490 | 0.982 |  | -0.015 | 0.794 |  | 0.157 |
| Torsion_Regional_global | 0.918 |  | 0.013 | 0.963 |  | -0.012 | 0.752 |  | -0.105 | 0.895 |  | -0.048 | 0.955 |  | -0.019 | 0.925 |  | 0.028 |
| Torsion_Basal_BA | 0.086 |  | -0.198 | 0.613 |  | 0.241 | 0.095 |  | 1.144 | 0.145 |  | 1.203 | 0.273 |  | 0.916 | 0.123 |  | 1.039 |
| Torsion_Basal_BAS | 0.552 |  | -0.055 | 0.862 |  | 0.076 | 0.474 |  | 0.525 | 0.371 |  | 0.794 | 0.351 |  | 0.812 | 0.148 |  | 1.028 |
| Torsion_Basal_BS | 0.545 |  | -0.056 | 0.994 |  | -0.003 | 0.126 |  | 0.854 | 0.128 |  | 0.932 | 0.230 |  | 0.677 | 0.144 |  | 0.712 |
| Torsion_Basal_BI | 0.936 |  | 0.009 | 0.914 |  | 0.041 | 0.409 |  | 0.501 | 0.526 |  | 0.428 | 0.961 |  | -0.031 | 0.862 |  | -0.089 |
| Torsion_Basal_BP | 0.393 |  | 0.084 | 0.765 |  | -0.120 | 0.870 |  | 0.106 | 0.901 |  | -0.083 | 0.611 |  | -0.330 | 0.446 |  | -0.413 |
| Torsion_Basal_BL | 0.772 |  | 0.030 | 0.181 |  | 0.451 | 0.013 | * | 1.120 | 0.029 | * | 1.169 | 0.009 | * | 1.261 | 0.002 | * | 1.252 |
| Torsion_Basal_MA | 0.118 |  | -0.195 | 0.696 |  | -0.162 | 0.584 |  | 0.321 | 0.500 |  | 0.444 | 0.880 |  | 0.099 | 0.876 |  | 0.090 |
| Torsion_Basal_MAS | 0.553 |  | -0.051 | 0.841 |  | -0.072 | 0.924 |  | 0.055 | 0.587 |  | 0.372 | 0.562 |  | 0.397 | 0.405 |  | 0.451 |
| Torsion_Basal_MS | 0.792 |  | -0.028 | 0.463 |  | -0.236 | 0.993 |  | -0.004 | 0.883 |  | 0.079 | 0.751 |  | -0.159 | 0.777 |  | -0.117 |
| Torsion_Basal_MI | 0.812 |  | 0.039 | 0.510 |  | 0.281 | 0.207 |  | 0.687 | 0.255 |  | 0.583 | 0.875 |  | 0.075 | 0.684 |  | -0.175 |
| Torsion_Basal_MP | 0.477 |  | 0.107 | 0.196 |  | 0.479 | 0.079 |  | 0.915 | 0.166 |  | 0.790 | 0.398 |  | 0.474 | 0.560 |  | 0.252 |
| Torsion_Basal_ML | 0.779 |  | -0.031 | 0.320 |  | 0.312 | 0.072 |  | 0.810 | 0.050 | * | 0.973 | 0.102 |  | 0.747 | 0.131 |  | 0.644 |
| Torsion_Basal_AA | 0.700 |  | -0.063 | 0.516 |  | -0.251 | 0.433 |  | -0.429 | 0.661 |  | -0.259 | 0.655 |  | -0.247 | 0.694 |  | -0.181 |
| Torsion_Basal_AS | 0.908 |  | -0.016 | 0.201 |  | -0.494 | 0.031 | * | -1.034 | 0.071 |  | -0.955 | 0.149 |  | -0.747 | 0.284 |  | -0.460 |
| Torsion_Basal_AI | 0.946 |  | -0.012 | 0.517 |  | 0.248 | 0.592 |  | 0.269 | 0.628 |  | 0.267 | 0.693 |  | 0.191 | 0.938 |  | -0.033 |
| Torsion_Basal_AL | 0.994 |  | 0.001 | 0.903 |  | 0.041 | 0.996 |  | -0.002 | 0.907 |  | 0.059 | 0.814 |  | 0.111 | 0.689 |  | 0.169 |
| Torsion_Basal_global | 0.773 |  | -0.028 | 0.844 |  | 0.051 | 0.289 |  | 0.364 | 0.255 |  | 0.425 | 0.461 |  | 0.267 | 0.402 |  | 0.260 |
| Strain_3D_BA | 0.991 |  | 0.011 | 0.834 |  | 0.562 | 0.668 |  | 1.793 | 0.138 |  | 6.633 | 0.104 |  | 6.323 | 0.134 |  | 4.505 |
| Strain_3D_BAS | 0.431 |  | 0.516 | 0.241 |  | 2.354 | 0.942 |  | -0.283 | 0.906 |  | 0.535 | 0.781 |  | 1.174 | 0.971 |  | 0.129 |
| Strain_3D_BS | 0.285 |  | 0.613 | 0.583 |  | 1.222 | 0.459 |  | -3.571 | 0.470 |  | -3.750 | 0.290 |  | -4.587 | 0.051 |  | -6.514 |
| Strain_3D_BI | 0.551 |  | 0.342 | 0.528 |  | 1.595 | 0.991 |  | -0.052 | 0.572 |  | 2.613 | 0.537 |  | 2.501 | 0.544 |  | -1.732 |
| Strain_3D_BP | 0.919 |  | -0.080 | 0.256 |  | -2.999 | 0.149 |  | -5.547 | 0.695 |  | -1.588 | 0.858 |  | 0.758 | 0.361 |  | -2.949 |
| Strain_3D_BL | 0.945 |  | -0.059 | 0.391 |  | -2.603 | 0.081 |  | -6.227 | 0.473 |  | -2.638 | 0.624 |  | -1.953 | 0.423 |  | -2.689 |
| Strain_3D_MA | 0.763 |  | 0.218 | 0.936 |  | -0.205 | 0.840 |  | -0.828 | 0.104 |  | 7.782 | 0.072 |  | 8.235 | 0.116 |  | 6.152 |
| Strain_3D_MAS | 0.324 |  | 0.529 | 0.770 |  | 0.791 | 0.177 |  | -6.983 | 0.752 |  | -1.912 | 0.635 |  | 2.649 | 0.927 |  | -0.432 |
| Strain_3D_MS | 0.102 |  | 1.029 | 0.139 |  | 3.754 | 0.497 |  | 3.223 | 0.402 |  | 4.830 | 0.456 |  | 4.079 | 0.940 |  | -0.314 |
| Strain_3D_MI | 0.552 |  | 0.400 | 0.141 |  | 3.380 | 0.031 | * | 8.831 | 0.035 | * | 12.654 | 0.044 | * | 11.195 | 0.116 |  | 5.919 |
| Strain_3D_MP | 0.542 |  | -0.444 | 0.319 |  | -2.191 | 0.980 |  | -0.097 | 0.283 |  | 5.853 | 0.192 |  | 6.416 | 0.340 |  | 3.512 |
| Strain_3D_ML | 0.783 |  | 0.202 | 0.348 |  | -2.768 | 0.153 |  | -5.583 | 0.867 |  | -0.758 | 0.875 |  | 0.721 | 0.978 |  | 0.111 |
| Strain_3D_AA | 0.894 |  | 0.108 | 0.845 |  | -0.539 | 0.321 |  | -5.465 | 0.957 |  | 0.400 | 0.530 |  | 4.787 | 0.569 |  | 3.084 |
| Strain_3D_AS | 0.574 |  | 0.421 | 0.364 |  | 2.176 | 0.835 |  | -0.973 | 0.644 |  | 2.800 | 0.417 |  | 5.860 | 0.584 |  | 2.414 |
| Strain_3D_AI | 0.663 |  | 0.371 | 0.347 |  | 1.871 | 0.913 |  | 0.519 | 0.462 |  | 4.390 | 0.301 |  | 5.711 | 0.454 |  | 2.860 |
| Strain_3D_AL | 0.842 |  | -0.151 | 0.999 |  | 0.003 | 0.273 |  | -5.283 | 0.696 |  | -2.145 | 0.951 |  | -0.319 | 0.751 |  | -1.375 |
| Strain_3D_global | 0.571 |  | 0.252 | 0.774 |  | 0.400 | 0.496 |  | -1.658 | 0.455 |  | 2.231 | 0.239 |  | 3.347 | 0.685 |  | 0.794 |
| RDisp_BA | 0.631 |  | -0.053 | 0.902 |  | 0.037 | 0.956 |  | 0.023 | 0.793 |  | -0.124 | 0.213 |  | -0.596 | 0.871 |  | -0.069 |
| RDisp_BAS | 0.030 | * | -0.218 | 0.005 | * | -0.969 | 0.001 | * | -1.533 | 0.000 | * | -2.031 | 0.000 | * | -2.131 | 0.005 | * | -1.346 |
| RDisp_BS | 0.018 | * | -0.320 | 0.006 | * | -0.856 | 0.018 | * | -1.063 | 0.009 | * | -1.284 | 0.041 | * | -0.975 | 0.648 |  | -0.206 |
| RDisp_BI | 0.258 |  | -0.209 | 0.869 |  | -0.061 | 0.312 |  | 0.473 | 0.105 |  | 0.818 | 0.043 | * | 1.008 | 0.008 | * | 1.320 |
| RDisp_BP | 0.497 |  | -0.129 | 0.060 |  | 0.740 | 0.000 | * | 1.887 | 0.000 | * | 2.600 | 0.000 | * | 2.425 | 0.000 | * | 2.291 |
| RDisp_BL | 0.720 |  | -0.050 | 0.017 | * | 0.731 | 0.002 | * | 1.406 | 0.001 | * | 1.675 | 0.027 | * | 1.133 | 0.002 | * | 1.417 |
| RDisp_MA | 0.986 |  | -0.002 | 0.979 |  | 0.007 | 0.843 |  | -0.084 | 0.782 |  | -0.147 | 0.398 |  | -0.467 | 0.745 |  | 0.150 |
| RDisp_MAS | 0.177 |  | -0.141 | 0.111 |  | -0.500 | 0.098 |  | -0.805 | 0.082 |  | -0.945 | 0.073 |  | -0.950 | 0.365 |  | -0.419 |
| RDisp_MS | 0.051 |  | -0.207 | 0.103 |  | -0.435 | 0.404 |  | -0.352 | 0.506 |  | -0.308 | 0.589 |  | -0.248 | 0.516 |  | 0.281 |
| RDisp_MI | 0.350 |  | -0.118 | 0.368 |  | -0.230 | 0.772 |  | -0.108 | 0.834 |  | 0.092 | 0.868 |  | -0.075 | 0.461 |  | 0.316 |
| RDisp_MP | 0.828 |  | -0.028 | 0.438 |  | 0.229 | 0.195 |  | 0.554 | 0.128 |  | 0.768 | 0.548 |  | 0.288 | 0.149 |  | 0.634 |
| RDisp_ML | 0.985 |  | 0.003 | 0.299 |  | 0.289 | 0.237 |  | 0.533 | 0.341 |  | 0.537 | 0.996 |  | -0.003 | 0.255 |  | 0.548 |
| RDisp_AA | 0.622 |  | 0.050 | 0.967 |  | 0.012 | 0.950 |  | 0.030 | 0.543 |  | 0.357 | 0.602 |  | 0.296 | 0.370 |  | 0.436 |
| RDisp_AS | 0.831 |  | 0.019 | 0.930 |  | -0.024 | 0.629 |  | 0.216 | 0.170 |  | 0.721 | 0.131 |  | 0.733 | 0.098 |  | 0.695 |
| RDisp_AI | 0.694 |  | 0.034 | 0.925 |  | -0.024 | 0.651 |  | 0.198 | 0.224 |  | 0.646 | 0.323 |  | 0.501 | 0.295 |  | 0.463 |
| RDisp_AL | 0.555 |  | 0.063 | 0.865 |  | 0.048 | 0.724 |  | 0.164 | 0.426 |  | 0.458 | 0.646 |  | 0.256 | 0.427 |  | 0.390 |
| RDisp_global | 0.426 |  | -0.082 | 0.750 |  | -0.063 | 0.738 |  | 0.095 | 0.458 |  | 0.239 | 0.812 |  | 0.075 | 0.156 |  | 0.431 |
| LDisp_BA | 0.398 |  | -0.092 | 0.351 |  | -0.316 | 0.028 | * | -1.225 | 0.003 | * | -1.991 | 0.002 | * | -1.998 | 0.142 |  | -0.762 |
| LDisp_BAS | 0.031 | * | -0.221 | 0.100 |  | -0.562 | 0.010 | * | -1.433 | 0.002 | * | -2.132 | 0.003 | * | -1.942 | 0.476 |  | -0.342 |
| LDisp_BS | 0.012 | * | -0.377 | 0.018 | * | -0.917 | 0.023 | * | -1.362 | 0.028 | * | -1.609 | 0.025 | * | -1.500 | 0.788 |  | 0.138 |
| LDisp_BI | 0.026 | * | -0.394 | 0.010 | * | -1.121 | 0.004 | * | -1.988 | 0.017 | * | -2.030 | 0.020 | * | -1.804 | 0.636 |  | -0.285 |
| LDisp_BP | 0.103 |  | -0.281 | 0.028 | * | -0.901 | 0.003 | * | -1.982 | 0.001 | * | -2.682 | 0.000 | * | -2.857 | 0.025 | * | -1.341 |
| LDisp_BL | 0.405 |  | -0.119 | 0.240 |  | -0.424 | 0.017 | * | -1.384 | 0.001 | * | -2.164 | 0.000 | * | -2.328 | 0.043 | * | -1.139 |
| LDisp_MA | 0.032 | * | -0.204 | 0.226 |  | -0.344 | 0.145 |  | -0.738 | 0.015 | * | -1.651 | 0.006 | * | -1.868 | 0.087 |  | -0.925 |
| LDisp_MAS | 0.001 | * | -0.258 | 0.014 | * | -0.697 | 0.039 | * | -1.002 | 0.005 | * | -1.863 | 0.004 | * | -1.883 | 0.118 |  | -0.684 |
| LDisp_MS | 0.001 | * | -0.260 | 0.005 | * | -0.766 | 0.025 | * | -1.079 | 0.034 | * | -1.399 | 0.081 |  | -1.056 | 0.676 |  | 0.160 |
| LDisp_MI | 0.085 |  | -0.156 | 0.025 | * | -0.602 | 0.003 | * | -1.252 | 0.031 | * | -1.231 | 0.065 |  | -0.919 | 0.875 |  | 0.058 |
| LDisp_MP | 0.348 |  | -0.087 | 0.317 |  | -0.306 | 0.027 | * | -0.989 | 0.020 | * | -1.361 | 0.017 | * | -1.408 | 0.274 |  | -0.539 |
| LDisp_ML | 0.155 |  | -0.130 | 0.703 |  | -0.115 | 0.244 |  | -0.589 | 0.051 |  | -1.283 | 0.032 | * | -1.466 | 0.306 |  | -0.608 |
| LDisp_AA | 0.140 |  | -0.104 | 0.308 |  | -0.240 | 0.675 |  | -0.158 | 0.188 |  | -0.715 | 0.096 |  | -0.889 | 0.513 |  | -0.249 |
| LDisp_AS | 0.164 |  | -0.092 | 0.136 |  | -0.325 | 0.381 |  | -0.326 | 0.174 |  | -0.763 | 0.215 |  | -0.657 | 0.863 |  | 0.063 |
| LDisp_AI | 0.368 |  | -0.068 | 0.759 |  | -0.059 | 0.693 |  | -0.132 | 0.324 |  | -0.463 | 0.374 |  | -0.409 | 0.486 |  | 0.252 |
| LDisp_AL | 0.194 |  | -0.093 | 0.934 |  | -0.019 | 0.886 |  | -0.051 | 0.355 |  | -0.455 | 0.216 |  | -0.633 | 0.900 |  | -0.052 |
| LDisp_global | 0.028 | * | -0.183 | 0.030 | * | -0.482 | 0.006 | * | -0.981 | 0.001 | * | -1.486 | 0.001 | * | -1.478 | 0.224 |  | -0.391 |
| X3DDisp_BA | 0.988 |  | 0.002 | 0.760 |  | -0.084 | 0.183 |  | 0.630 | 0.008 | * | 1.595 | 0.002 | * | 1.909 | 0.234 |  | 0.613 |
| X3DDisp_BAS | 0.268 |  | 0.126 | 0.169 |  | 0.365 | 0.012 | * | 1.228 | 0.000 | * | 2.338 | 0.000 | * | 2.398 | 0.224 |  | 0.612 |
| X3DDisp_BS | 0.668 |  | -0.080 | 0.095 |  | 0.556 | 0.065 |  | 1.042 | 0.042 | * | 1.419 | 0.027 | * | 1.468 | 0.538 |  | -0.326 |
| X3DDisp_BI | 0.549 |  | -0.139 | 0.497 |  | 0.246 | 0.269 |  | 0.597 | 0.492 |  | 0.449 | 0.367 |  | 0.547 | 0.122 |  | -0.782 |
| X3DDisp_BP | 0.688 |  | -0.087 | 0.803 |  | -0.092 | 0.691 |  | 0.238 | 0.445 |  | 0.528 | 0.128 |  | 0.995 | 0.647 |  | -0.257 |
| X3DDisp_BL | 0.691 |  | -0.067 | 0.346 |  | -0.284 | 0.916 |  | 0.053 | 0.206 |  | 0.717 | 0.021 | * | 1.250 | 0.885 |  | 0.072 |
| X3DDisp_MA | 0.725 |  | -0.046 | 0.802 |  | 0.059 | 0.452 |  | 0.320 | 0.072 |  | 1.086 | 0.010 | * | 1.639 | 0.224 |  | 0.591 |
| X3DDisp_MAS | 0.729 |  | 0.040 | 0.157 |  | 0.293 | 0.087 |  | 0.635 | 0.004 | * | 1.492 | 0.001 | * | 1.849 | 0.070 |  | 0.656 |
| X3DDisp_MS | 0.701 |  | -0.050 | 0.527 |  | 0.131 | 0.805 |  | 0.086 | 0.966 |  | 0.020 | 0.409 |  | 0.426 | 0.274 |  | -0.415 |
| X3DDisp_MI | 0.442 |  | -0.109 | 0.524 |  | 0.135 | 0.756 |  | 0.110 | 0.653 |  | -0.217 | 0.757 |  | 0.148 | 0.235 |  | -0.463 |
| X3DDisp_MP | 0.453 |  | -0.111 | 0.643 |  | -0.130 | 0.825 |  | 0.102 | 0.737 |  | 0.189 | 0.206 |  | 0.725 | 0.908 |  | -0.055 |
| X3DDisp_ML | 0.468 |  | -0.100 | 0.511 |  | -0.177 | 0.812 |  | -0.107 | 0.498 |  | 0.407 | 0.094 |  | 1.055 | 0.760 |  | 0.156 |
| X3DDisp_AA | 0.726 |  | -0.035 | 0.786 |  | 0.058 | 0.961 |  | 0.016 | 0.599 |  | 0.242 | 0.125 |  | 0.733 | 0.379 |  | 0.286 |
| X3DDisp_AS | 0.883 |  | 0.013 | 0.718 |  | -0.067 | 0.547 |  | -0.200 | 0.469 |  | -0.322 | 0.840 |  | 0.083 | 0.862 |  | -0.045 |
| X3DDisp_AI | 0.833 |  | -0.020 | 0.785 |  | -0.052 | 0.540 |  | -0.211 | 0.207 |  | -0.556 | 0.654 |  | -0.184 | 0.349 |  | -0.302 |
| X3DDisp_AL | 0.624 |  | -0.053 | 0.648 |  | -0.101 | 0.837 |  | -0.069 | 0.925 |  | -0.044 | 0.411 |  | 0.419 | 0.848 |  | 0.076 |
| X3DDisp_global | 0.723 |  | -0.045 | 0.735 |  | 0.053 | 0.334 |  | 0.278 | 0.111 |  | 0.584 | 0.009 | * | 0.966 | 0.930 |  | 0.025 |
| AreaTracking_BA | 0.645 |  | 0.469 | 0.005 | * | 9.089 | 0.000 | * | 21.352 | 0.000 | * | 28.859 | 0.001 | * | 27.690 | 0.003 | * | 17.656 |
| AreaTracking_BAS | 0.489 |  | 0.889 | 0.034 | * | 7.964 | 0.005 | * | 17.842 | 0.002 | * | 27.163 | 0.002 | * | 28.070 | 0.026 | * | 15.975 |
| AreaTracking_BS | 0.234 |  | 1.695 | 0.326 |  | 3.679 | 0.364 |  | 4.415 | 0.096 |  | 9.165 | 0.023 | * | 13.843 | 0.077 |  | 9.674 |
| AreaTracking_BI | 0.284 |  | 1.385 | 0.792 |  | 0.823 | 0.511 |  | -3.315 | 0.488 |  | -4.229 | 0.555 |  | -3.781 | 0.273 |  | -6.419 |
| AreaTracking_BP | 0.171 |  | 2.272 | 0.013 | * | 9.843 | 0.008 | * | 19.392 | 0.007 | * | 26.807 | 0.006 | * | 28.850 | 0.167 |  | 11.248 |
| AreaTracking_BL | 0.140 |  | 1.582 | 0.000 | * | 15.485 | 0.000 | * | 36.241 | 0.000 | * | 54.162 | 0.000 | * | 59.621 | 0.000 | * | 40.681 |
| AreaTracking_MA | 0.962 |  | 0.055 | 0.375 |  | 2.846 | 0.048 | * | 11.818 | 0.022 | * | 19.647 | 0.016 | * | 23.359 | 0.082 |  | 11.311 |
| AreaTracking_MAS | 0.105 |  | 2.595 | 0.039 | * | 8.462 | 0.009 | * | 20.660 | 0.002 | * | 31.272 | 0.001 | * | 35.019 | 0.041 | * | 16.845 |
| AreaTracking_MS | 0.057 |  | 2.581 | 0.051 |  | 8.029 | 0.135 |  | 10.988 | 0.146 |  | 12.867 | 0.080 |  | 15.454 | 0.686 |  | 2.848 |
| AreaTracking_MI | 0.231 |  | 1.555 | 0.066 |  | 6.493 | 0.210 |  | 8.055 | 0.882 |  | 1.248 | 0.748 |  | 2.831 | 0.632 |  | -3.340 |
| AreaTracking_MP | 0.502 |  | 1.194 | 0.067 |  | 7.482 | 0.093 |  | 12.314 | 0.179 |  | 13.105 | 0.056 |  | 19.831 | 0.392 |  | 6.847 |
| AreaTracking_ML | 0.929 |  | -0.114 | 0.168 |  | 4.942 | 0.021 | * | 16.487 | 0.005 | * | 28.049 | 0.001 | * | 36.575 | 0.010 | * | 17.768 |
| AreaTracking_AA | 0.480 |  | 1.700 | 0.231 |  | 8.871 | 0.112 |  | 17.474 | 0.049 | * | 41.313 | 0.034 | * | 59.231 | 0.132 |  | 26.792 |
| AreaTracking_AS | 0.209 |  | 1.828 | 0.242 |  | 6.772 | 0.314 |  | 8.929 | 0.218 |  | 20.781 | 0.163 |  | 29.572 | 0.688 |  | 4.495 |
| AreaTracking_AI | 0.575 |  | 1.000 | 0.165 |  | 9.690 | 0.128 |  | 17.896 | 0.132 |  | 23.945 | 0.125 |  | 28.261 | 0.583 |  | 8.173 |
| AreaTracking_AL | 0.597 |  | 1.342 | 0.444 |  | 7.181 | 0.322 |  | 18.321 | 0.146 |  | 39.152 | 0.092 |  | 58.087 | 0.249 |  | 31.070 |
| AreaTracking_global | 0.280 |  | 1.376 | 0.016 | * | 7.353 | 0.002 | * | 14.928 | 0.000 | * | 23.331 | 0.000 | * | 28.907 | 0.020 | * | 13.225 |

| **VARIABLES** | **P-val7** | **Sig7** | **Contr-HCM7** | **P-val8** | **Sig8** | **Contr-HCM8** | **P-val9** | **Sig9** | **Contr-HCM9** | **P-val10** | **Sig10** | **Contr-HCM10** | **P-val11** | **Sig11** | **Contr-HCM11** | **P-val12** | **Sig12** | **Contr-HCM12** |
| --- | --- | --- | --- | --- | --- | --- | --- | --- | --- | --- | --- | --- | --- | --- | --- | --- | --- | --- |
| Volume.mL. | 0.000 | * | -50.063 | 0.000 | * | -47.194 | 0.000 | * | -44.701 | 0.000 | * | -43.888 | 0.000 | * | -42.021 | 0.000 | * | -39.967 |
| Myo.Vol_.mL. | 0.000 | * | -24.957 | 0.000 | * | -24.117 | 0.000 | * | -23.346 | 0.000 | * | -23.005 | 0.000 | * | -22.226 | 0.000 | * | -21.332 |
| RStrain_BA | 0.334 |  | 2.503 | 0.958 |  | 0.129 | 0.556 |  | -1.430 | 0.527 |  | -1.377 | 0.944 |  | -0.121 | 0.391 |  | 0.818 |
| RStrain_BAS | 0.215 |  | 4.266 | 0.414 |  | 2.595 | 0.733 |  | 0.966 | 0.852 |  | 0.474 | 0.711 |  | 0.707 | 0.485 |  | 0.628 |
| RStrain_BS | 0.435 |  | -2.053 | 0.451 |  | -1.778 | 0.227 |  | -2.596 | 0.170 |  | -2.611 | 0.279 |  | -1.452 | 0.857 |  | -0.104 |
| RStrain_BI | 0.269 |  | -2.775 | 0.170 |  | -2.863 | 0.112 |  | -2.924 | 0.086 |  | -2.834 | 0.145 |  | -1.727 | 0.877 |  | -0.076 |
| RStrain_BP | 0.095 |  | -4.111 | 0.144 |  | -3.381 | 0.117 |  | -3.375 | 0.132 |  | -3.065 | 0.156 |  | -2.263 | 0.974 |  | -0.026 |
| RStrain_BL | 0.036 | * | -6.107 | 0.014 | * | -6.102 | 0.004 | * | -7.023 | 0.007 | * | -5.977 | 0.026 | * | -3.641 | 0.829 |  | -0.179 |
| RStrain_MA | 0.928 |  | 0.245 | 0.629 |  | -1.198 | 0.233 |  | -2.628 | 0.239 |  | -2.384 | 0.467 |  | -1.225 | 0.743 |  | -0.256 |
| RStrain_MAS | 0.374 |  | -3.761 | 0.366 |  | -3.138 | 0.261 |  | -3.229 | 0.237 |  | -3.096 | 0.367 |  | -1.921 | 0.521 |  | -0.657 |
| RStrain_MS | 0.679 |  | -1.233 | 0.735 |  | 0.962 | 0.916 |  | 0.247 | 0.963 |  | -0.094 | 0.876 |  | -0.241 | 0.867 |  | -0.105 |
| RStrain_MI | 0.543 |  | 1.241 | 0.650 |  | 0.842 | 0.839 |  | 0.316 | 0.865 |  | 0.253 | 0.761 |  | 0.410 | 0.555 |  | 0.344 |
| RStrain_MP | 0.685 |  | -1.062 | 0.755 |  | -0.732 | 0.756 |  | -0.603 | 0.747 |  | -0.588 | 0.837 |  | -0.338 | 0.766 |  | -0.230 |
| RStrain_ML | 0.235 |  | -3.790 | 0.144 |  | -3.983 | 0.050 |  | -4.489 | 0.043 | * | -4.198 | 0.075 |  | -2.855 | 0.230 |  | -0.839 |
| RStrain_AA | 0.642 |  | -2.201 | 0.229 |  | -4.062 | 0.042 | * | -5.820 | 0.027 | * | -5.519 | 0.067 |  | -3.608 | 0.139 |  | -1.248 |
| RStrain_AS | 0.963 |  | 0.343 | 0.519 |  | -3.048 | 0.075 |  | -5.876 | 0.027 | * | -6.280 | 0.021 | * | -5.228 | 0.057 |  | -1.869 |
| RStrain_AI | 0.828 |  | -0.695 | 0.343 |  | -2.666 | 0.117 |  | -4.083 | 0.081 |  | -4.021 | 0.084 |  | -2.966 | 0.254 |  | -0.864 |
| RStrain_AL | 0.368 |  | -2.840 | 0.382 |  | -2.484 | 0.143 |  | -3.683 | 0.075 |  | -3.884 | 0.155 |  | -2.600 | 0.219 |  | -0.925 |
| RStrain_global | 0.440 |  | -1.377 | 0.207 |  | -1.930 | 0.045 | * | -2.889 | 0.033 | * | -2.826 | 0.093 |  | -1.817 | 0.479 |  | -0.349 |
| CStrain_BA | 0.107 |  | 5.619 | 0.062 |  | 5.349 | 0.064 |  | 4.716 | 0.114 |  | 3.876 | 0.291 |  | 2.036 | 0.885 |  | -0.129 |
| CStrain_BAS | 0.237 |  | 4.764 | 0.238 |  | 3.785 | 0.221 |  | 3.982 | 0.278 |  | 3.609 | 0.473 |  | 2.077 | 0.655 |  | -0.591 |
| CStrain_BS | 0.305 |  | 3.122 | 0.160 |  | 3.689 | 0.043 | * | 5.350 | 0.036 | * | 5.662 | 0.067 |  | 4.258 | 0.541 |  | 0.700 |
| CStrain_BI | 0.055 |  | -6.298 | 0.165 |  | -3.853 | 0.631 |  | -1.211 | 0.948 |  | -0.158 | 0.922 |  | 0.212 | 0.774 |  | -0.283 |
| CStrain_BP | 0.529 |  | -3.023 | 0.825 |  | -0.768 | 0.855 |  | 0.580 | 0.765 |  | 0.909 | 0.601 |  | 1.322 | 0.671 |  | 0.569 |
| CStrain_BL | 0.079 |  | 7.674 | 0.009 | * | 8.376 | 0.002 | * | 9.652 | 0.002 | * | 8.996 | 0.008 | * | 6.600 | 0.229 |  | 1.438 |
| CStrain_MA | 0.524 |  | 1.710 | 0.300 |  | 2.494 | 0.132 |  | 3.408 | 0.148 |  | 2.998 | 0.195 |  | 1.977 | 0.693 |  | 0.302 |
| CStrain_MAS | 0.259 |  | 3.425 | 0.067 |  | 4.273 | 0.029 | * | 5.437 | 0.038 | * | 5.236 | 0.078 |  | 3.887 | 0.439 |  | 0.918 |
| CStrain_MS | 0.831 |  | -0.557 | 0.436 |  | 1.839 | 0.167 |  | 3.289 | 0.161 |  | 3.166 | 0.250 |  | 2.071 | 0.922 |  | 0.082 |
| CStrain_MI | 0.145 |  | -3.934 | 0.561 |  | -1.248 | 0.829 |  | 0.417 | 0.802 |  | 0.462 | 0.825 |  | 0.363 | 0.791 |  | 0.196 |
| CStrain_MP | 0.676 |  | -1.400 | 0.579 |  | 1.437 | 0.148 |  | 3.642 | 0.148 |  | 3.532 | 0.118 |  | 3.029 | 0.248 |  | 1.321 |
| CStrain_ML | 0.536 |  | 2.058 | 0.171 |  | 3.887 | 0.009 | * | 6.588 | 0.009 | * | 6.195 | 0.019 | * | 4.525 | 0.372 |  | 0.946 |
| CStrain_AA | 0.535 |  | -2.723 | 0.952 |  | -0.237 | 0.326 |  | 3.467 | 0.248 |  | 3.724 | 0.248 |  | 2.858 | 0.807 |  | 0.314 |
| CStrain_AS | 0.268 |  | -3.469 | 0.902 |  | 0.370 | 0.195 |  | 3.946 | 0.171 |  | 4.005 | 0.224 |  | 2.821 | 0.734 |  | 0.353 |
| CStrain_AI | 0.350 |  | -3.481 | 0.866 |  | -0.519 | 0.311 |  | 2.721 | 0.312 |  | 2.476 | 0.485 |  | 1.413 | 0.915 |  | 0.110 |
| CStrain_AL | 0.405 |  | -3.865 | 0.987 |  | 0.062 | 0.188 |  | 4.943 | 0.140 |  | 5.180 | 0.152 |  | 3.929 | 0.571 |  | 0.766 |
| CStrain_global | 0.992 |  | -0.024 | 0.351 |  | 1.808 | 0.058 |  | 3.808 | 0.059 |  | 3.743 | 0.106 |  | 2.712 | 0.627 |  | 0.439 |
| LStrain_BA | 0.086 |  | 2.996 | 0.047 | * | 3.087 | 0.071 |  | 2.632 | 0.161 |  | 1.897 | 0.365 |  | 1.009 | 0.470 |  | 0.345 |
| LStrain_BAS | 0.904 |  | -0.226 | 0.866 |  | -0.271 | 0.860 |  | -0.257 | 0.656 |  | -0.624 | 0.525 |  | -0.726 | 0.410 |  | -0.396 |
| LStrain_BS | 0.689 |  | -0.859 | 0.260 |  | -2.012 | 0.298 |  | -1.749 | 0.314 |  | -1.628 | 0.449 |  | -0.982 | 0.746 |  | -0.208 |
| LStrain_BI | 0.881 |  | -0.351 | 0.708 |  | -0.749 | 0.914 |  | -0.196 | 0.994 |  | -0.012 | 0.917 |  | 0.133 | 0.993 |  | 0.006 |
| LStrain_BP | 0.632 |  | 1.104 | 0.603 |  | 1.061 | 0.469 |  | 1.311 | 0.526 |  | 1.031 | 0.552 |  | 0.734 | 0.424 |  | 0.507 |
| LStrain_BL | 0.008 | * | 6.061 | 0.013 | * | 5.066 | 0.019 | * | 3.979 | 0.052 |  | 2.797 | 0.135 |  | 1.634 | 0.108 |  | 0.790 |
| LStrain_MA | 0.666 |  | -1.142 | 0.826 |  | -0.412 | 0.537 |  | 1.152 | 0.597 |  | 0.922 | 0.650 |  | 0.665 | 0.358 |  | 0.669 |
| LStrain_MAS | 0.385 |  | 2.228 | 0.140 |  | 2.588 | 0.077 |  | 3.017 | 0.151 |  | 2.426 | 0.359 |  | 1.401 | 0.358 |  | 0.693 |
| LStrain_MS | 0.961 |  | 0.123 | 0.510 |  | 1.393 | 0.318 |  | 1.963 | 0.436 |  | 1.430 | 0.697 |  | 0.578 | 0.854 |  | 0.137 |
| LStrain_MI | 0.882 |  | -0.336 | 0.658 |  | 0.760 | 0.621 |  | 0.829 | 0.849 |  | 0.306 | 0.812 |  | 0.317 | 0.612 |  | 0.380 |
| LStrain_MP | 0.872 |  | 0.365 | 0.786 |  | 0.448 | 0.433 |  | 1.242 | 0.593 |  | 0.801 | 0.508 |  | 0.814 | 0.369 |  | 0.605 |
| LStrain_ML | 0.493 |  | -1.857 | 0.437 |  | -1.485 | 0.995 |  | 0.012 | 0.914 |  | -0.180 | 0.942 |  | -0.096 | 0.702 |  | 0.243 |
| LStrain_AA | 0.433 |  | 2.304 | 0.113 |  | 4.230 | 0.017 | * | 6.475 | 0.028 | * | 5.770 | 0.043 | * | 4.244 | 0.296 |  | 1.118 |
| LStrain_AS | 0.395 |  | -1.730 | 0.911 |  | 0.229 | 0.486 |  | 1.361 | 0.549 |  | 1.093 | 0.787 |  | 0.378 | 0.692 |  | -0.255 |
| LStrain_AI | 0.919 |  | 0.283 | 0.472 |  | 1.786 | 0.221 |  | 2.678 | 0.267 |  | 2.177 | 0.464 |  | 1.092 | 0.964 |  | -0.029 |
| LStrain_AL | 0.389 |  | 3.514 | 0.081 |  | 5.656 | 0.012 | * | 7.601 | 0.017 | * | 6.819 | 0.025 | * | 4.921 | 0.264 |  | 1.107 |
| LStrain_global | 0.567 |  | 0.780 | 0.211 |  | 1.335 | 0.086 |  | 2.003 | 0.173 |  | 1.564 | 0.306 |  | 1.006 | 0.510 |  | 0.357 |
| Rotation_BA | 0.813 |  | -0.254 | 0.310 |  | 0.874 | 0.203 |  | 0.973 | 0.240 |  | 0.810 | 0.212 |  | 0.656 | 0.065 |  | 0.376 |
| Rotation_BAS | 0.074 |  | -1.686 | 0.264 |  | -0.933 | 0.240 |  | -0.891 | 0.188 |  | -0.930 | 0.140 |  | -0.794 | 0.660 |  | -0.095 |
| Rotation_BS | 0.679 |  | 0.382 | 0.229 |  | 0.848 | 0.268 |  | 0.677 | 0.336 |  | 0.529 | 0.503 |  | 0.297 | 0.795 |  | -0.059 |
| Rotation_BI | 0.229 |  | -1.072 | 0.713 |  | -0.289 | 0.976 |  | 0.023 | 0.960 |  | 0.037 | 0.966 |  | -0.025 | 0.747 |  | 0.093 |
| Rotation_BP | 0.001 | * | -3.168 | 0.002 | * | -2.231 | 0.011 | * | -1.771 | 0.026 | * | -1.503 | 0.043 | * | -1.112 | 0.245 |  | -0.277 |
| Rotation_BL | 0.007 | * | -3.271 | 0.035 | * | -1.904 | 0.086 |  | -1.349 | 0.134 |  | -1.072 | 0.344 |  | -0.539 | 0.743 |  | 0.080 |
| Rotation_MA | 0.144 |  | -1.398 | 0.455 |  | -0.571 | 0.895 |  | -0.091 | 0.977 |  | -0.018 | 0.796 |  | 0.128 | 0.473 |  | 0.143 |
| Rotation_MAS | 0.040 | * | -1.787 | 0.100 |  | -1.232 | 0.108 |  | -1.025 | 0.202 |  | -0.713 | 0.522 |  | -0.259 | 0.546 |  | 0.109 |
| Rotation_MS | 0.448 |  | -0.603 | 0.916 |  | -0.058 | 0.731 |  | 0.175 | 0.407 |  | 0.376 | 0.178 |  | 0.479 | 0.319 |  | 0.165 |
| Rotation_MI | 0.016 | * | -1.863 | 0.096 |  | -1.115 | 0.108 |  | -0.942 | 0.203 |  | -0.695 | 0.339 |  | -0.391 | 0.804 |  | -0.057 |
| Rotation_MP | 0.001 | * | -2.567 | 0.014 | * | -1.695 | 0.018 | * | -1.580 | 0.037 | * | -1.286 | 0.105 |  | -0.795 | 0.716 |  | -0.097 |
| Rotation_ML | 0.005 | * | -2.362 | 0.067 |  | -1.173 | 0.212 |  | -0.738 | 0.446 |  | -0.401 | 0.912 |  | 0.042 | 0.100 |  | 0.266 |
| Rotation_AA | 0.015 | * | -3.409 | 0.020 | * | -2.866 | 0.099 |  | -1.833 | 0.205 |  | -1.337 | 0.599 |  | -0.437 | 0.972 |  | 0.011 |
| Rotation_AS | 0.023 | * | -3.079 | 0.064 |  | -2.270 | 0.206 |  | -1.425 | 0.413 |  | -0.852 | 0.945 |  | -0.057 | 0.933 |  | 0.027 |
| Rotation_AI | 0.016 | * | -3.622 | 0.024 | * | -2.994 | 0.047 | * | -2.311 | 0.083 |  | -1.866 | 0.269 |  | -0.919 | 0.572 |  | -0.196 |
| Rotation_AL | 0.013 | * | -3.523 | 0.035 | * | -2.627 | 0.108 |  | -1.873 | 0.226 |  | -1.365 | 0.539 |  | -0.557 | 0.996 |  | -0.002 |
| Rotation_global | 0.003 | * | -2.081 | 0.028 | * | -1.264 | 0.098 |  | -0.874 | 0.176 |  | -0.643 | 0.444 |  | -0.269 | 0.834 |  | 0.030 |
| Twist_BA | 0.076 |  | 0.790 | 0.153 |  | 0.514 | 0.295 |  | 0.296 | 0.461 |  | 0.193 | 0.829 |  | 0.048 | 0.808 |  | -0.025 |
| Twist_BAS | 0.055 |  | 1.024 | 0.103 |  | 0.713 | 0.057 |  | 0.704 | 0.029 | * | 0.754 | 0.025 | * | 0.614 | 0.134 |  | 0.164 |
| Twist_BS | 0.209 |  | 0.532 | 0.218 |  | 0.429 | 0.172 |  | 0.380 | 0.154 |  | 0.367 | 0.232 |  | 0.250 | 0.809 |  | 0.025 |
| Twist_BI | 0.685 |  | -0.167 | 0.553 |  | -0.213 | 0.125 |  | -0.426 | 0.108 |  | -0.402 | 0.100 |  | -0.338 | 0.134 |  | -0.173 |
| Twist_BP | 0.307 |  | -0.403 | 0.383 |  | -0.285 | 0.354 |  | -0.271 | 0.400 |  | -0.227 | 0.323 |  | -0.209 | 0.193 |  | -0.118 |
| Twist_BL | 0.003 | * | 0.863 | 0.003 | * | 0.761 | 0.007 | * | 0.603 | 0.006 | * | 0.559 | 0.016 | * | 0.382 | 0.134 |  | 0.111 |
| Twist_MA | 0.865 |  | -0.194 | 0.402 |  | -0.777 | 0.392 |  | -0.648 | 0.465 |  | -0.522 | 0.539 |  | -0.385 | 0.465 |  | -0.227 |
| Twist_MAS | 0.465 |  | 0.821 | 0.741 |  | 0.316 | 0.528 |  | 0.512 | 0.253 |  | 0.907 | 0.102 |  | 1.084 | 0.197 |  | 0.355 |
| Twist_MS | 0.702 |  | -0.385 | 0.613 |  | -0.419 | 0.884 |  | -0.099 | 0.717 |  | 0.232 | 0.397 |  | 0.448 | 0.355 |  | 0.240 |
| Twist_MI | 0.377 |  | -0.920 | 0.274 |  | -1.002 | 0.090 |  | -1.343 | 0.139 |  | -1.094 | 0.253 |  | -0.670 | 0.319 |  | -0.299 |
| Twist_MP | 0.825 |  | 0.177 | 0.709 |  | 0.231 | 0.886 |  | -0.088 | 0.985 |  | -0.011 | 0.827 |  | 0.109 | 0.824 |  | 0.053 |
| Twist_ML | 0.069 |  | 1.653 | 0.055 |  | 1.376 | 0.040 | * | 1.117 | 0.035 | * | 1.140 | 0.061 |  | 0.898 | 0.195 |  | 0.282 |
| Twist_AA | 0.289 |  | -1.690 | 0.078 |  | -2.479 | 0.173 |  | -1.698 | 0.355 |  | -1.129 | 0.757 |  | -0.323 | 0.694 |  | -0.186 |
| Twist_AS | 0.117 |  | -2.335 | 0.094 |  | -2.160 | 0.304 |  | -1.219 | 0.651 |  | -0.525 | 0.767 |  | 0.290 | 0.682 |  | 0.178 |
| Twist_AI | 0.167 |  | -2.157 | 0.090 |  | -2.302 | 0.092 |  | -2.144 | 0.159 |  | -1.768 | 0.418 |  | -0.829 | 0.506 |  | -0.304 |
| Twist_AL | 0.868 |  | 0.261 | 0.895 |  | -0.162 | 0.999 |  | -0.001 | 0.850 |  | 0.212 | 0.702 |  | 0.382 | 0.878 |  | 0.070 |
| Twist_global | 0.837 |  | -0.133 | 0.521 |  | -0.343 | 0.560 |  | -0.270 | 0.861 |  | -0.082 | 0.790 |  | 0.109 | 0.966 |  | 0.009 |
| Torsion_Regional_BA | 0.730 |  | 0.192 | 0.895 |  | -0.059 | 0.759 |  | -0.116 | 0.731 |  | -0.123 | 0.670 |  | -0.132 | 0.738 |  | -0.046 |
| Torsion_Regional_BAS | 0.379 |  | 0.490 | 0.628 |  | 0.231 | 0.469 |  | 0.297 | 0.277 |  | 0.420 | 0.158 |  | 0.442 | 0.307 |  | 0.130 |
| Torsion_Regional_BS | 0.489 |  | 0.290 | 0.533 |  | 0.232 | 0.453 |  | 0.228 | 0.378 |  | 0.248 | 0.361 |  | 0.212 | 0.529 |  | 0.066 |
| Torsion_Regional_BI | 0.689 |  | -0.166 | 0.509 |  | -0.250 | 0.177 |  | -0.427 | 0.214 |  | -0.364 | 0.292 |  | -0.248 | 0.577 |  | -0.068 |
| Torsion_Regional_BP | 0.918 |  | -0.040 | 0.889 |  | -0.045 | 0.691 |  | -0.122 | 0.792 |  | -0.078 | 0.813 |  | -0.057 | 0.699 |  | -0.038 |
| Torsion_Regional_BL | 0.023 | * | 0.813 | 0.033 | * | 0.670 | 0.049 | * | 0.533 | 0.037 | * | 0.543 | 0.054 |  | 0.420 | 0.150 |  | 0.135 |
| Torsion_Regional_MA | 0.112 |  | -0.800 | 0.017 | * | -1.059 | 0.046 | * | -0.762 | 0.123 |  | -0.564 | 0.412 |  | -0.243 | 0.613 |  | -0.069 |
| Torsion_Regional_MAS | 0.247 |  | -0.401 | 0.150 |  | -0.503 | 0.323 |  | -0.306 | 0.719 |  | -0.108 | 0.542 |  | 0.150 | 0.732 |  | 0.037 |
| Torsion_Regional_MS | 0.027 | * | -0.840 | 0.008 | * | -0.843 | 0.032 | * | -0.631 | 0.169 |  | -0.386 | 0.815 |  | -0.056 | 0.495 |  | 0.069 |
| Torsion_Regional_MI | 0.380 |  | -0.348 | 0.206 |  | -0.461 | 0.280 |  | -0.389 | 0.398 |  | -0.286 | 0.874 |  | -0.041 | 0.911 |  | 0.012 |
| Torsion_Regional_MP | 0.509 |  | 0.296 | 0.773 |  | 0.114 | 0.945 |  | -0.024 | 0.965 |  | -0.014 | 0.686 |  | 0.112 | 0.467 |  | 0.108 |
| Torsion_Regional_ML | 0.709 |  | -0.191 | 0.374 |  | -0.360 | 0.412 |  | -0.290 | 0.554 |  | -0.208 | 0.811 |  | -0.072 | 0.974 |  | -0.005 |
| Torsion_Regional_AA | 0.295 |  | -0.583 | 0.270 |  | -0.649 | 0.305 |  | -0.484 | 0.327 |  | -0.445 | 0.484 |  | -0.284 | 0.478 |  | -0.130 |
| Torsion_Regional_AS | 0.442 |  | -0.510 | 0.372 |  | -0.545 | 0.567 |  | -0.255 | 0.519 |  | -0.271 | 0.551 |  | -0.222 | 0.303 |  | -0.159 |
| Torsion_Regional_AI | 0.785 |  | -0.147 | 0.560 |  | -0.275 | 0.919 |  | -0.043 | 0.924 |  | -0.039 | 0.835 |  | 0.073 | 0.969 |  | 0.006 |
| Torsion_Regional_AL | 0.402 |  | -0.479 | 0.343 |  | -0.493 | 0.613 |  | -0.239 | 0.566 |  | -0.265 | 0.662 |  | -0.166 | 0.442 |  | -0.110 |
| Torsion_Regional_global | 0.549 |  | -0.151 | 0.227 |  | -0.268 | 0.356 |  | -0.190 | 0.570 |  | -0.120 | 0.979 |  | -0.005 | 0.967 |  | -0.004 |
| Torsion_Basal_BA | 0.113 |  | 0.827 | 0.254 |  | 0.480 | 0.516 |  | 0.225 | 0.698 |  | 0.125 | 0.999 |  | 0.000 | 0.762 |  | -0.034 |
| Torsion_Basal_BAS | 0.131 |  | 0.846 | 0.232 |  | 0.572 | 0.132 |  | 0.626 | 0.069 |  | 0.709 | 0.050 | * | 0.613 | 0.235 |  | 0.151 |
| Torsion_Basal_BS | 0.161 |  | 0.550 | 0.165 |  | 0.468 | 0.131 |  | 0.406 | 0.124 |  | 0.379 | 0.189 |  | 0.265 | 0.743 |  | 0.030 |
| Torsion_Basal_BI | 0.974 |  | 0.014 | 0.833 |  | -0.081 | 0.314 |  | -0.304 | 0.264 |  | -0.295 | 0.258 |  | -0.234 | 0.374 |  | -0.093 |
| Torsion_Basal_BP | 0.454 |  | -0.316 | 0.466 |  | -0.266 | 0.362 |  | -0.295 | 0.433 |  | -0.233 | 0.407 |  | -0.200 | 0.262 |  | -0.108 |
| Torsion_Basal_BL | 0.005 | * | 0.908 | 0.012 | * | 0.723 | 0.038 | * | 0.543 | 0.037 | * | 0.509 | 0.082 |  | 0.333 | 0.370 |  | 0.078 |
| Torsion_Basal_MA | 0.744 |  | -0.159 | 0.276 |  | -0.434 | 0.275 |  | -0.374 | 0.348 |  | -0.307 | 0.473 |  | -0.204 | 0.597 |  | -0.069 |
| Torsion_Basal_MAS | 0.671 |  | 0.188 | 0.980 |  | -0.010 | 0.807 |  | 0.083 | 0.475 |  | 0.238 | 0.214 |  | 0.348 | 0.328 |  | 0.114 |
| Torsion_Basal_MS | 0.847 |  | -0.066 | 0.733 |  | -0.102 | 0.921 |  | -0.025 | 0.762 |  | 0.072 | 0.484 |  | 0.139 | 0.419 |  | 0.074 |
| Torsion_Basal_MI | 0.549 |  | -0.215 | 0.387 |  | -0.290 | 0.153 |  | -0.428 | 0.228 |  | -0.344 | 0.409 |  | -0.188 | 0.682 |  | -0.047 |
| Torsion_Basal_MP | 0.689 |  | 0.120 | 0.725 |  | 0.085 | 0.899 |  | -0.031 | 0.999 |  | 0.000 | 0.838 |  | 0.042 | 0.803 |  | 0.025 |
| Torsion_Basal_ML | 0.117 |  | 0.567 | 0.148 |  | 0.424 | 0.122 |  | 0.365 | 0.094 |  | 0.397 | 0.118 |  | 0.331 | 0.233 |  | 0.112 |
| Torsion_Basal_AA | 0.278 |  | -0.416 | 0.068 |  | -0.655 | 0.164 |  | -0.453 | 0.337 |  | -0.317 | 0.736 |  | -0.097 | 0.775 |  | -0.037 |
| Torsion_Basal_AS | 0.169 |  | -0.472 | 0.123 |  | -0.478 | 0.322 |  | -0.280 | 0.667 |  | -0.122 | 0.773 |  | 0.070 | 0.732 |  | 0.036 |
| Torsion_Basal_AI | 0.342 |  | -0.328 | 0.180 |  | -0.425 | 0.165 |  | -0.426 | 0.245 |  | -0.356 | 0.547 |  | -0.150 | 0.756 |  | -0.034 |
| Torsion_Basal_AL | 0.916 |  | 0.040 | 0.783 |  | -0.085 | 0.899 |  | -0.036 | 0.951 |  | 0.019 | 0.773 |  | 0.077 | 0.802 |  | 0.031 |
| Torsion_Basal_global | 0.610 |  | 0.132 | 0.988 |  | -0.003 | 0.896 |  | -0.026 | 0.878 |  | 0.030 | 0.670 |  | 0.072 | 0.859 |  | 0.014 |
| Strain_3D_BA | 0.096 |  | 3.756 | 0.575 |  | 1.146 | 0.817 |  | -0.454 | 0.619 |  | -0.920 | 0.718 |  | -0.544 | 0.720 |  | 0.315 |
| Strain_3D_BAS | 0.640 |  | 1.477 | 0.877 |  | 0.460 | 0.839 |  | -0.548 | 0.757 |  | -0.742 | 0.932 |  | -0.154 | 0.734 |  | 0.286 |
| Strain_3D_BS | 0.187 |  | -3.388 | 0.199 |  | -2.965 | 0.109 |  | -3.449 | 0.091 |  | -3.282 | 0.179 |  | -1.864 | 0.655 |  | -0.249 |
| Strain_3D_BI | 0.501 |  | -1.686 | 0.362 |  | -1.943 | 0.274 |  | -2.025 | 0.220 |  | -2.065 | 0.324 |  | -1.231 | 0.855 |  | 0.098 |
| Strain_3D_BP | 0.164 |  | -3.544 | 0.239 |  | -2.738 | 0.213 |  | -2.685 | 0.195 |  | -2.625 | 0.201 |  | -2.004 | 0.980 |  | -0.019 |
| Strain_3D_BL | 0.145 |  | -3.542 | 0.070 |  | -3.867 | 0.020 | * | -4.760 | 0.026 | * | -4.113 | 0.080 |  | -2.446 | 0.956 |  | 0.045 |
| Strain_3D_MA | 0.390 |  | 2.707 | 0.793 |  | 0.703 | 0.441 |  | -1.684 | 0.335 |  | -1.925 | 0.423 |  | -1.289 | 0.423 |  | -0.579 |
| Strain_3D_MAS | 0.715 |  | -1.494 | 0.706 |  | -1.250 | 0.491 |  | -1.833 | 0.400 |  | -1.993 | 0.478 |  | -1.399 | 0.537 |  | -0.608 |
| Strain_3D_MS | 0.803 |  | -0.792 | 0.664 |  | 1.229 | 0.772 |  | 0.659 | 0.894 |  | 0.265 | 0.940 |  | -0.113 | 0.775 |  | -0.170 |
| Strain_3D_MI | 0.520 |  | 1.566 | 0.659 |  | 0.961 | 0.639 |  | 0.832 | 0.729 |  | 0.553 | 0.769 |  | 0.387 | 0.765 |  | 0.169 |
| Strain_3D_MP | 0.806 |  | 0.674 | 0.854 |  | 0.425 | 0.833 |  | 0.393 | 0.883 |  | 0.259 | 0.922 |  | 0.159 | 0.788 |  | -0.204 |
| Strain_3D_ML | 0.523 |  | -2.069 | 0.359 |  | -2.426 | 0.172 |  | -2.817 | 0.130 |  | -2.777 | 0.173 |  | -1.936 | 0.233 |  | -0.784 |
| Strain_3D_AA | 0.872 |  | -0.597 | 0.520 |  | -2.003 | 0.062 |  | -4.893 | 0.035 | * | -4.815 | 0.064 |  | -3.359 | 0.134 |  | -1.235 |
| Strain_3D_AS | 0.816 |  | -0.836 | 0.379 |  | -2.771 | 0.061 |  | -4.976 | 0.031 | * | -5.321 | 0.029 | * | -4.512 | 0.085 |  | -1.613 |
| Strain_3D_AI | 0.916 |  | 0.293 | 0.694 |  | -0.980 | 0.262 |  | -2.517 | 0.141 |  | -2.940 | 0.102 |  | -2.520 | 0.251 |  | -0.847 |
| Strain_3D_AL | 0.637 |  | -1.437 | 0.881 |  | -0.433 | 0.414 |  | -2.001 | 0.182 |  | -2.699 | 0.147 |  | -2.284 | 0.178 |  | -0.900 |
| Strain_3D_global | 0.723 |  | -0.557 | 0.449 |  | -1.029 | 0.095 |  | -2.047 | 0.055 |  | -2.195 | 0.107 |  | -1.570 | 0.403 |  | -0.393 |
| RDisp_BA | 0.096 |  | 0.654 | 0.389 |  | 0.280 | 0.855 |  | -0.060 | 0.683 |  | -0.140 | 0.498 |  | -0.204 | 0.894 |  | -0.021 |
| RDisp_BAS | 0.136 |  | -0.532 | 0.060 |  | -0.564 | 0.013 | * | -0.762 | 0.032 | * | -0.699 | 0.111 |  | -0.478 | 0.739 |  | -0.048 |
| RDisp_BS | 0.663 |  | 0.184 | 0.730 |  | 0.133 | 0.580 |  | -0.209 | 0.501 |  | -0.261 | 0.677 |  | -0.143 | 0.693 |  | 0.070 |
| RDisp_BI | 0.008 | * | 1.282 | 0.026 | * | 0.914 | 0.145 |  | 0.559 | 0.269 |  | 0.421 | 0.471 |  | 0.243 | 0.677 |  | 0.086 |
| RDisp_BP | 0.000 | * | 2.073 | 0.001 | * | 1.319 | 0.036 | * | 0.737 | 0.121 |  | 0.540 | 0.372 |  | 0.278 | 0.867 |  | 0.033 |
| RDisp_BL | 0.000 | * | 1.800 | 0.003 | * | 1.160 | 0.079 |  | 0.622 | 0.242 |  | 0.419 | 0.592 |  | 0.168 | 0.780 |  | 0.048 |
| RDisp_MA | 0.039 | * | 0.829 | 0.216 |  | 0.419 | 0.941 |  | -0.025 | 0.714 |  | -0.129 | 0.435 |  | -0.237 | 0.434 |  | -0.127 |
| RDisp_MAS | 0.910 |  | 0.038 | 0.648 |  | -0.140 | 0.218 |  | -0.407 | 0.279 |  | -0.358 | 0.346 |  | -0.266 | 0.499 |  | -0.092 |
| RDisp_MS | 0.488 |  | 0.233 | 0.909 |  | 0.035 | 0.794 |  | -0.079 | 0.805 |  | -0.073 | 0.957 |  | -0.014 | 0.813 |  | 0.030 |
| RDisp_MI | 0.515 |  | 0.245 | 0.963 |  | -0.015 | 0.909 |  | -0.034 | 0.844 |  | -0.055 | 0.717 |  | -0.082 | 0.709 |  | -0.047 |
| RDisp_MP | 0.094 |  | 0.718 | 0.452 |  | 0.252 | 0.760 |  | -0.093 | 0.574 |  | -0.165 | 0.397 |  | -0.207 | 0.425 |  | -0.106 |
| RDisp_ML | 0.025 | * | 1.055 | 0.173 |  | 0.508 | 0.869 |  | 0.057 | 0.846 |  | -0.068 | 0.540 |  | -0.184 | 0.552 |  | -0.101 |
| RDisp_AA | 0.131 |  | 0.561 | 0.790 |  | 0.094 | 0.570 |  | -0.192 | 0.530 |  | -0.208 | 0.500 |  | -0.182 | 0.498 |  | -0.091 |
| RDisp_AS | 0.176 |  | 0.460 | 0.789 |  | 0.089 | 0.855 |  | -0.057 | 0.924 |  | -0.028 | 0.984 |  | -0.005 | 0.911 |  | -0.012 |
| RDisp_AI | 0.441 |  | 0.280 | 0.641 |  | -0.150 | 0.365 |  | -0.262 | 0.351 |  | -0.245 | 0.308 |  | -0.204 | 0.240 |  | -0.109 |
| RDisp_AL | 0.228 |  | 0.471 | 0.830 |  | -0.073 | 0.341 |  | -0.301 | 0.286 |  | -0.327 | 0.231 |  | -0.296 | 0.249 |  | -0.146 |
| RDisp_global | 0.023 | * | 0.647 | 0.253 |  | 0.266 | 0.894 |  | -0.032 | 0.730 |  | -0.086 | 0.613 |  | -0.113 | 0.749 |  | -0.040 |
| LDisp_BA | 0.554 |  | 0.294 | 0.499 |  | 0.233 | 0.518 |  | -0.235 | 0.548 |  | -0.209 | 0.557 |  | -0.169 | 0.372 |  | -0.120 |
| LDisp_BAS | 0.193 |  | 0.575 | 0.159 |  | 0.444 | 0.925 |  | 0.032 | 0.973 |  | 0.011 | 0.980 |  | 0.007 | 0.577 |  | -0.068 |
| LDisp_BS | 0.066 |  | 0.797 | 0.129 |  | 0.513 | 0.761 |  | 0.098 | 0.816 |  | 0.071 | 0.867 |  | 0.044 | 0.785 |  | -0.039 |
| LDisp_BI | 0.554 |  | 0.262 | 0.704 |  | 0.141 | 0.721 |  | -0.127 | 0.795 |  | -0.088 | 0.662 |  | -0.128 | 0.545 |  | -0.106 |
| LDisp_BP | 0.719 |  | -0.162 | 0.911 |  | -0.037 | 0.270 |  | -0.376 | 0.357 |  | -0.299 | 0.295 |  | -0.278 | 0.377 |  | -0.138 |
| LDisp_BL | 0.842 |  | 0.105 | 0.758 |  | 0.111 | 0.181 |  | -0.484 | 0.214 |  | -0.418 | 0.199 |  | -0.342 | 0.365 |  | -0.123 |
| LDisp_MA | 0.910 |  | -0.048 | 0.778 |  | -0.085 | 0.111 |  | -0.465 | 0.099 |  | -0.445 | 0.090 |  | -0.360 | 0.228 |  | -0.132 |
| LDisp_MAS | 0.650 |  | 0.147 | 0.783 |  | 0.070 | 0.339 |  | -0.240 | 0.215 |  | -0.285 | 0.184 |  | -0.234 | 0.261 |  | -0.097 |
| LDisp_MS | 0.032 | * | 0.559 | 0.155 |  | 0.314 | 0.891 |  | 0.031 | 0.939 |  | -0.015 | 0.909 |  | 0.018 | 0.934 |  | 0.006 |
| LDisp_MI | 0.202 |  | 0.373 | 0.199 |  | 0.316 | 0.479 |  | 0.162 | 0.467 |  | 0.153 | 0.459 |  | 0.128 | 0.511 |  | 0.056 |
| LDisp_MP | 0.816 |  | 0.086 | 0.580 |  | 0.153 | 0.458 |  | -0.194 | 0.551 |  | -0.143 | 0.718 |  | -0.066 | 0.771 |  | 0.022 |
| LDisp_ML | 0.614 |  | 0.230 | 0.585 |  | 0.177 | 0.250 |  | -0.348 | 0.240 |  | -0.325 | 0.335 |  | -0.206 | 0.960 |  | 0.005 |
| LDisp_AA | 0.231 |  | 0.325 | 0.338 |  | 0.230 | 0.890 |  | -0.030 | 0.695 |  | -0.075 | 0.404 |  | -0.115 | 0.814 |  | -0.014 |
| LDisp_AS | 0.078 |  | 0.471 | 0.099 |  | 0.383 | 0.549 |  | 0.120 | 0.752 |  | 0.053 | 0.972 |  | 0.004 | 0.460 |  | 0.033 |
| LDisp_AI | 0.038 | * | 0.539 | 0.031 | * | 0.479 | 0.315 |  | 0.196 | 0.377 |  | 0.148 | 0.344 |  | 0.114 | 0.179 |  | 0.067 |
| LDisp_AL | 0.167 |  | 0.377 | 0.260 |  | 0.260 | 0.919 |  | -0.021 | 0.767 |  | -0.054 | 0.763 |  | -0.040 | 0.661 |  | 0.020 |
| LDisp_global | 0.245 |  | 0.307 | 0.234 |  | 0.232 | 0.575 |  | -0.118 | 0.545 |  | -0.119 | 0.521 |  | -0.102 | 0.625 |  | -0.039 |
| X3DDisp_BA | 0.189 |  | -0.683 | 0.175 |  | -0.453 | 0.735 |  | 0.128 | 0.723 |  | 0.134 | 0.877 |  | 0.052 | 0.444 |  | -0.140 |
| X3DDisp_BAS | 0.225 |  | -0.554 | 0.234 |  | -0.364 | 0.610 |  | 0.186 | 0.598 |  | 0.194 | 0.727 |  | 0.115 | 0.527 |  | -0.104 |
| X3DDisp_BS | 0.017 | * | -1.155 | 0.029 | * | -0.825 | 0.745 |  | -0.134 | 0.740 |  | -0.134 | 0.579 |  | -0.198 | 0.087 |  | -0.361 |
| X3DDisp_BI | 0.004 | * | -1.298 | 0.015 | * | -0.904 | 0.366 |  | -0.365 | 0.339 |  | -0.370 | 0.309 |  | -0.351 | 0.087 |  | -0.408 |
| X3DDisp_BP | 0.012 | * | -1.291 | 0.012 | * | -0.947 | 0.397 |  | -0.336 | 0.392 |  | -0.325 | 0.443 |  | -0.249 | 0.217 |  | -0.265 |
| X3DDisp_BL | 0.022 | * | -1.258 | 0.008 | * | -0.983 | 0.505 |  | -0.261 | 0.547 |  | -0.232 | 0.546 |  | -0.199 | 0.266 |  | -0.214 |
| X3DDisp_MA | 0.330 |  | -0.427 | 0.514 |  | -0.189 | 0.466 |  | 0.235 | 0.513 |  | 0.203 | 0.674 |  | 0.109 | 0.544 |  | -0.096 |
| X3DDisp_MAS | 0.663 |  | -0.130 | 0.815 |  | 0.055 | 0.244 |  | 0.343 | 0.361 |  | 0.258 | 0.608 |  | 0.122 | 0.384 |  | -0.118 |
| X3DDisp_MS | 0.031 | * | -0.576 | 0.245 |  | -0.278 | 0.767 |  | 0.085 | 0.907 |  | 0.031 | 0.684 |  | -0.087 | 0.045 | * | -0.271 |
| X3DDisp_MI | 0.056 |  | -0.565 | 0.135 |  | -0.374 | 0.677 |  | -0.116 | 0.641 |  | -0.116 | 0.545 |  | -0.118 | 0.086 |  | -0.221 |
| X3DDisp_MP | 0.099 |  | -0.661 | 0.092 |  | -0.480 | 0.872 |  | -0.048 | 0.922 |  | -0.028 | 0.921 |  | 0.021 | 0.408 |  | -0.112 |
| X3DDisp_ML | 0.083 |  | -0.795 | 0.118 |  | -0.485 | 0.879 |  | 0.051 | 0.872 |  | 0.052 | 0.929 |  | 0.023 | 0.425 |  | -0.124 |
| X3DDisp_AA | 0.358 |  | -0.214 | 0.618 |  | 0.116 | 0.323 |  | 0.255 | 0.296 |  | 0.258 | 0.235 |  | 0.240 | 0.572 |  | 0.066 |
| X3DDisp_AS | 0.392 |  | -0.177 | 0.973 |  | -0.007 | 0.551 |  | 0.136 | 0.510 |  | 0.134 | 0.460 |  | 0.123 | 0.891 |  | 0.013 |
| X3DDisp_AI | 0.155 |  | -0.331 | 0.590 |  | -0.114 | 0.838 |  | 0.046 | 0.733 |  | 0.068 | 0.579 |  | 0.085 | 0.848 |  | -0.017 |
| X3DDisp_AL | 0.255 |  | -0.292 | 0.895 |  | 0.031 | 0.426 |  | 0.204 | 0.381 |  | 0.218 | 0.281 |  | 0.216 | 0.658 |  | 0.050 |
| X3DDisp_global | 0.027 | * | -0.650 | 0.071 |  | -0.388 | 0.926 |  | 0.025 | 0.934 |  | 0.021 | 0.976 |  | -0.007 | 0.298 |  | -0.145 |
| AreaTracking_BA | 0.042 | * | 8.375 | 0.015 | * | 8.235 | 0.019 | * | 7.294 | 0.062 |  | 5.563 | 0.231 |  | 2.875 | 0.992 |  | -0.011 |
| AreaTracking_BAS | 0.301 |  | 5.535 | 0.312 |  | 4.158 | 0.296 |  | 4.245 | 0.404 |  | 3.484 | 0.622 |  | 1.784 | 0.583 |  | -0.901 |
| AreaTracking_BS | 0.497 |  | 2.884 | 0.580 |  | 1.893 | 0.262 |  | 3.911 | 0.215 |  | 4.418 | 0.240 |  | 3.568 | 0.800 |  | 0.414 |
| AreaTracking_BI | 0.105 |  | -7.618 | 0.167 |  | -5.347 | 0.585 |  | -1.937 | 0.845 |  | -0.652 | 0.974 |  | 0.093 | 0.846 |  | -0.297 |
| AreaTracking_BP | 0.938 |  | -0.452 | 0.627 |  | 2.169 | 0.409 |  | 3.581 | 0.428 |  | 3.196 | 0.386 |  | 2.845 | 0.450 |  | 1.423 |
| AreaTracking_BL | 0.000 | * | 16.830 | 0.000 | * | 16.067 | 0.000 | * | 15.923 | 0.000 | * | 13.427 | 0.002 | * | 9.037 | 0.140 |  | 2.286 |
| AreaTracking_MA | 0.892 |  | -0.635 | 0.724 |  | 1.289 | 0.284 |  | 3.828 | 0.308 |  | 3.370 | 0.370 |  | 2.412 | 0.443 |  | 1.052 |
| AreaTracking_MAS | 0.265 |  | 6.740 | 0.063 |  | 7.608 | 0.035 | * | 8.921 | 0.062 |  | 7.974 | 0.144 |  | 5.541 | 0.379 |  | 1.707 |
| AreaTracking_MS | 0.988 |  | 0.075 | 0.375 |  | 3.948 | 0.175 |  | 6.070 | 0.207 |  | 5.253 | 0.345 |  | 3.055 | 0.846 |  | 0.296 |
| AreaTracking_MI | 0.317 |  | -4.548 | 0.872 |  | -0.549 | 0.672 |  | 1.404 | 0.764 |  | 0.958 | 0.733 |  | 0.923 | 0.639 |  | 0.644 |
| AreaTracking_MP | 0.972 |  | -0.209 | 0.486 |  | 2.936 | 0.149 |  | 5.980 | 0.182 |  | 5.323 | 0.143 |  | 4.593 | 0.219 |  | 2.180 |
| AreaTracking_ML | 1.000 |  | -0.001 | 0.528 |  | 2.626 | 0.063 |  | 7.210 | 0.075 |  | 6.612 | 0.107 |  | 4.932 | 0.360 |  | 1.492 |
| AreaTracking_AA | 0.900 |  | -1.151 | 0.516 |  | 5.253 | 0.093 |  | 12.848 | 0.085 |  | 12.077 | 0.096 |  | 8.803 | 0.456 |  | 1.917 |
| AreaTracking_AS | 0.265 |  | -6.658 | 0.944 |  | 0.429 | 0.286 |  | 6.401 | 0.264 |  | 6.236 | 0.357 |  | 3.926 | 0.938 |  | 0.145 |
| AreaTracking_AI | 0.611 |  | -4.257 | 0.812 |  | 1.641 | 0.228 |  | 7.280 | 0.211 |  | 6.607 | 0.334 |  | 3.833 | 0.887 |  | 0.257 |
| AreaTracking_AL | 0.941 |  | 0.916 | 0.395 |  | 7.762 | 0.058 |  | 16.546 | 0.050 | * | 15.635 | 0.054 |  | 11.269 | 0.327 |  | 2.548 |
| AreaTracking_global | 0.791 |  | 0.989 | 0.216 |  | 3.757 | 0.041 | * | 6.844 | 0.059 |  | 6.217 | 0.114 |  | 4.343 | 0.524 |  | 0.947 |

| **Supplementary Table 2. Univariate ANOVAs (for first 10 PCs) and MANOVAs for each of the 12 homologous time frames performed on PC scores coming from GPA in SSS + LS + PCA procedure.** | | | | | | | | | | | |
| --- | --- | --- | --- | --- | --- | --- | --- | --- | --- | --- | --- |
|  |  |  |  |  |  |  |  |  |  |  |  |
| **Time frame 1** | R-sq multiv | P-value multiv | R-sq | P-value | Sig univ | **Time frame 7** | R-sq multiv | P-value multiv | R-sq | P-value | Sig univ |
| PC1 | 0.036 | 0.026 | 0.038 | 0.115 |  | PC1 | 0.071 | 0.010 | 0.093 | 0.013 | * |
| PC2 | NA | NA | 0.000 | 0.890 |  | PC2 | NA | NA | 0.033 | 0.142 |  |
| PC3 | NA | NA | 0.023 | 0.214 |  | PC3 | NA | NA | 0.013 | 0.378 |  |
| PC4 | NA | NA | 0.018 | 0.293 |  | PC4 | NA | NA | 0.055 | 0.049 | * |
| PC5 | NA | NA | 0.034 | 0.129 |  | PC5 | NA | NA | 0.003 | 0.647 |  |
| PC6 | NA | NA | 0.177 | 0.002 | * | PC6 | NA | NA | 0.041 | 0.104 |  |
| PC7 | NA | NA | 0.002 | 0.748 |  | PC7 | NA | NA | 0.020 | 0.259 |  |
| PC8 | NA | NA | 0.030 | 0.156 |  | PC8 | NA | NA | 0.072 | 0.034 | * |
| PC9 | NA | NA | 0.020 | 0.246 |  | PC9 | NA | NA | 0.017 | 0.310 |  |
| PC10 | NA | NA | 0.024 | 0.220 |  | PC10 | NA | NA | 0.011 | 0.414 |  |
| **Time frame 2** | R-sq multiv | P-value multiv | R-sq | P-value | Sig univ | **Time frame 8** | R-sq multiv | P-value multiv | R-sq | P-value | Sig univ |
| PC1 | 0.030 | 0.054 | 0.005 | 0.562 |  | PC1 | 0.033 | 0.075 | 0.061 | 0.050 |  |
| PC2 | NA | NA | 0.012 | 0.343 |  | PC2 | NA | NA | 0.000 | 0.950 |  |
| PC3 | NA | NA | 0.042 | 0.095 |  | PC3 | NA | NA | 0.002 | 0.741 |  |
| PC4 | NA | NA | 0.111 | 0.006 | * | PC4 | NA | NA | 0.008 | 0.474 |  |
| PC5 | NA | NA | 0.000 | 0.897 |  | PC5 | NA | NA | 0.000 | 0.855 |  |
| PC6 | NA | NA | 0.132 | 0.007 | * | PC6 | NA | NA | 0.006 | 0.505 |  |
| PC7 | NA | NA | 0.099 | 0.014 | * | PC7 | NA | NA | 0.010 | 0.437 |  |
| PC8 | NA | NA | 0.008 | 0.432 |  | PC8 | NA | NA | 0.042 | 0.116 |  |
| PC9 | NA | NA | 0.001 | 0.819 |  | PC9 | NA | NA | 0.001 | 0.809 |  |
| PC10 | NA | NA | 0.011 | 0.395 |  | PC10 | NA | NA | 0.008 | 0.466 |  |
| **Time frame 3** | R-sq multiv | P-value multiv | R-sq | P-value | Sig univ | **Time frame 9** | R-sq multiv | P-value multiv | R-sq | P-value | Sig univ |
| PC1 | 0.051 | 0.007 | 0.071 | 0.035 | * | PC1 | 0.016 | 0.331 | 0.003 | 0.675 |  |
| PC2 | NA | NA | 0.088 | 0.016 | * | PC2 | NA | NA | 0.044 | 0.084 |  |
| PC3 | NA | NA | 0.008 | 0.459 |  | PC3 | NA | NA | 0.001 | 0.817 |  |
| PC4 | NA | NA | 0.046 | 0.084 |  | PC4 | NA | NA | 0.000 | 0.951 |  |
| PC5 | NA | NA | 0.027 | 0.201 |  | PC5 | NA | NA | 0.000 | 0.906 |  |
| PC6 | NA | NA | 0.020 | 0.264 |  | PC6 | NA | NA | 0.090 | 0.020 | * |
| PC7 | NA | NA | 0.104 | 0.010 | * | PC7 | NA | NA | 0.069 | 0.039 | * |
| PC8 | NA | NA | 0.029 | 0.159 |  | PC8 | NA | NA | 0.037 | 0.111 |  |
| PC9 | NA | NA | 0.017 | 0.287 |  | PC9 | NA | NA | 0.030 | 0.158 |  |
| PC10 | NA | NA | 0.022 | 0.225 |  | PC10 | NA | NA | 0.002 | 0.748 |  |
| **Time frame 4** | R-sq multiv | P-value multiv | R-sq | P-value | Sig univ | **Time frame 10** | R-sq multiv | P-value multiv | R-sq | P-value | Sig univ |
| PC1 | 0.049 | 0.002 | 0.117 | 0.005 | * | PC1 | 0.019 | 0.238 | 0.004 | 0.611 |  |
| PC2 | NA | NA | 0.003 | 0.657 |  | PC2 | NA | NA | 0.049 | 0.062 |  |
| PC3 | NA | NA | 0.006 | 0.520 |  | PC3 | NA | NA | 0.000 | 0.964 |  |
| PC4 | NA | NA | 0.001 | 0.836 |  | PC4 | NA | NA | 0.000 | 0.947 |  |
| PC5 | NA | NA | 0.017 | 0.296 |  | PC5 | NA | NA | 0.003 | 0.650 |  |
| PC6 | NA | NA | 0.158 | 0.001 | * | PC6 | NA | NA | 0.130 | 0.004 | * |
| PC7 | NA | NA | 0.044 | 0.094 |  | PC7 | NA | NA | 0.059 | 0.041 | * |
| PC8 | NA | NA | 0.070 | 0.028 | * | PC8 | NA | NA | 0.022 | 0.213 |  |
| PC9 | NA | NA | 0.031 | 0.191 |  | PC9 | NA | NA | 0.051 | 0.053 |  |
| PC10 | NA | NA | 0.005 | 0.592 |  | PC10 | NA | NA | 0.009 | 0.442 |  |
| **Time frame 5** | R-sq multiv | P-value multiv | R-sq | P-value | Sig univ | **Time frame 11** | R-sq multiv | P-value multiv | R-sq | P-value | Sig univ |
| PC1 | 0.060 | 0.001 | 0.166 | 0.003 | * | PC1 | 0.026 | 0.114 | 0.013 | 0.320 |  |
| PC2 | NA | NA | 0.006 | 0.480 |  | PC2 | NA | NA | 0.019 | 0.286 |  |
| PC3 | NA | NA | 0.016 | 0.295 |  | PC3 | NA | NA | 0.004 | 0.598 |  |
| PC4 | NA | NA | 0.004 | 0.622 |  | PC4 | NA | NA | 0.000 | 0.975 |  |
| PC5 | NA | NA | 0.010 | 0.433 |  | PC5 | NA | NA | 0.025 | 0.192 |  |
| PC6 | NA | NA | 0.179 | 0.001 | * | PC6 | NA | NA | 0.144 | 0.003 | * |
| PC7 | NA | NA | 0.008 | 0.462 |  | PC7 | NA | NA | 0.051 | 0.073 |  |
| PC8 | NA | NA | 0.050 | 0.064 |  | PC8 | NA | NA | 0.024 | 0.228 |  |
| PC9 | NA | NA | 0.035 | 0.134 |  | PC9 | NA | NA | 0.084 | 0.017 | * |
| PC10 | NA | NA | 0.001 | 0.750 |  | PC10 | NA | NA | 0.041 | 0.094 |  |
| **Time frame 6** | R-sq multiv | P-value multiv | R-sq | P-value | Sig univ | **Time frame 12** | R-sq multiv | P-value multiv | R-sq | P-value | Sig univ |
| PC1 | 0.027 | 0.078 | 0.001 | 0.834 |  | PC1 | 0.041 | 0.014 | 0.054 | 0.047 | * |
| PC2 | NA | NA | 0.001 | 0.768 |  | PC2 | NA | NA | 0.000 | 0.941 |  |
| PC3 | NA | NA | 0.043 | 0.082 |  | PC3 | NA | NA | 0.030 | 0.184 |  |
| PC4 | NA | NA | 0.043 | 0.082 |  | PC4 | NA | NA | 0.001 | 0.785 |  |
| PC5 | NA | NA | 0.001 | 0.847 |  | PC5 | NA | NA | 0.037 | 0.109 |  |
| PC6 | NA | NA | 0.197 | 0.001 | * | PC6 | NA | NA | 0.161 | 0.002 | * |
| PC7 | NA | NA | 0.021 | 0.256 |  | PC7 | NA | NA | 0.024 | 0.193 |  |
| PC8 | NA | NA | 0.003 | 0.654 |  | PC8 | NA | NA | 0.043 | 0.108 |  |
| PC9 | NA | NA | 0.022 | 0.211 |  | PC9 | NA | NA | 0.076 | 0.028 | * |
| PC10 | NA | NA | 0.011 | 0.380 |  | PC10 | NA | NA | 0.016 | 0.314 |  |

| **Supplementary Table 3. Univariate Correlations between per-individual mean centered 3DSTE variables and PC1, PC2 and PC3 coming from GPA in SSS + LS in SSS + PCA procedure. For each PC 3DSTE variable are ordered in increasing order according to correlation coefficient.** | | | | | | | | | | | |
| --- | --- | --- | --- | --- | --- | --- | --- | --- | --- | --- | --- |
| **PC1** | **cor** | **P-value** | **Sig** | **PC2** | **cor** | **P-value** | **Sig** | **PC3** | **cor** | **P-value** | **Sig** |
| LDisp_BAS | -0.939 | 0.001 | * | CStrain_AA | -0.568 | 0.001 | * | CStrain_BI | -0.453 | 0.001 | * |
| RDisp_global | -0.933 | 0.001 | * | AreaTracking_AS | -0.566 | 0.001 | * | CStrain_BS | -0.400 | 0.001 | * |
| LDisp_BA | -0.929 | 0.001 | * | AreaTracking_AA | -0.542 | 0.001 | * | Rotation_BP | -0.362 | 0.001 | * |
| LDisp_BL | -0.928 | 0.001 | * | CStrain_AS | -0.528 | 0.001 | * | LDisp_MI | -0.309 | 0.001 | * |
| LDisp_BS | -0.926 | 0.001 | * | CStrain_AI | -0.511 | 0.001 | * | Rotation_BL | -0.298 | 0.001 | * |
| LDisp_global | -0.912 | 0.001 | * | CStrain_AL | -0.496 | 0.001 | * | AreaTracking_BS | -0.289 | 0.001 | * |
| LDisp_BP | -0.911 | 0.001 | * | AreaTracking_AI | -0.495 | 0.001 | * | LDisp_BI | -0.273 | 0.001 | * |
| RDisp_ML | -0.908 | 0.001 | * | LStrain_AS | -0.479 | 0.001 | * | Rotation_BI | -0.270 | 0.001 | * |
| RDisp_MP | -0.896 | 0.001 | * | AreaTracking_AL | -0.464 | 0.001 | * | LDisp_MP | -0.270 | 0.001 | * |
| RDisp_BL | -0.893 | 0.001 | * | LStrain_AA | -0.385 | 0.001 | * | RDisp_AI | -0.254 | 0.001 | * |
| RDisp_MA | -0.889 | 0.001 | * | LStrain_AI | -0.330 | 0.001 | * | Rotation_BAS | -0.247 | 0.001 | * |
| LDisp_BI | -0.882 | 0.001 | * | CStrain_MA | -0.325 | 0.001 | * | LDisp_BP | -0.238 | 0.001 | * |
| RDisp_BA | -0.874 | 0.001 | * | LStrain_AL | -0.315 | 0.001 | * | Strain_3D_MA | -0.235 | 0.001 | * |
| LDisp_MA | -0.866 | 0.001 | * | Strain_3D_AS | -0.295 | 0.001 | * | Rotation_BS | -0.231 | 0.001 | * |
| RDisp_BP | -0.855 | 0.001 | * | RStrain_AS | -0.274 | 0.001 | * | AreaTracking_BI | -0.208 | 0.001 | * |
| RDisp_MI | -0.854 | 0.001 | * | CStrain_global | -0.260 | 0.001 | * | CStrain_BAS | -0.195 | 0.001 | * |
| LDisp_ML | -0.854 | 0.001 | * | Rotation_AS | -0.259 | 0.001 | * | AreaTracking_BAS | -0.193 | 0.001 | * |
| RDisp_BI | -0.824 | 0.001 | * | Strain_3D_AA | -0.256 | 0.001 | * | RStrain_MA | -0.187 | 0.001 | * |
| LDisp_MAS | -0.823 | 0.001 | * | Torsion_Regional_AL | -0.251 | 0.001 | * | LDisp_AI | -0.185 | 0.001 | * |
| LDisp_MP | -0.815 | 0.001 | * | Torsion_Regional_AS | -0.251 | 0.001 | * | Rotation_MS | -0.183 | 0.001 | * |
| LDisp_MS | -0.775 | 0.001 | * | CStrain_BS | -0.247 | 0.001 | * | RDisp_AS | -0.176 | 0.001 | * |
| LDisp_MI | -0.763 | 0.001 | * | X3DDisp_AL | -0.234 | 0.001 | * | CStrain_MAS | -0.161 | 0.001 | * |
| RDisp_BS | -0.726 | 0.001 | * | X3DDisp_AS | -0.231 | 0.001 | * | LDisp_MS | -0.160 | 0.001 | * |
| RDisp_BAS | -0.714 | 0.001 | * | LDisp_BI | -0.231 | 0.001 | * | Rotation_BA | -0.154 | 0.001 | * |
| Rotation_MI | -0.705 | 0.001 | * | X3DDisp_AA | -0.231 | 0.001 | * | RStrain_AI | -0.149 | 0.001 | * |
| RDisp_AL | -0.704 | 0.001 | * | X3DDisp_AI | -0.228 | 0.001 | * | Strain_3D_ML | -0.142 | 0.001 | * |
| Rotation_BI | -0.690 | 0.001 | * | Rotation_MAS | -0.223 | 0.001 | * | LDisp_global | -0.142 | 0.001 | * |
| RDisp_MAS | -0.690 | 0.001 | * | Rotation_AA | -0.220 | 0.001 | * | AreaTracking_MAS | -0.141 | 0.001 | * |
| RDisp_MS | -0.631 | 0.001 | * | Rotation_BP | -0.194 | 0.001 | * | LDisp_ML | -0.137 | 0.001 | * |
| LDisp_AL | -0.606 | 0.001 | * | CStrain_MS | -0.186 | 0.001 | * | Strain_3D_MP | -0.136 | 0.002 | * |
| Rotation_MP | -0.577 | 0.001 | * | AreaTracking_global | -0.185 | 0.001 | * | LDisp_AL | -0.129 | 0.001 | * |
| RDisp_AA | -0.577 | 0.001 | * | CStrain_ML | -0.185 | 0.001 | * | Strain_3D_MAS | -0.127 | 0.001 | * |
| LDisp_AA | -0.560 | 0.001 | * | RStrain_AA | -0.184 | 0.001 | * | Rotation_MP | -0.120 | 0.002 | * |
| RStrain_global | -0.558 | 0.001 | * | Rotation_BI | -0.178 | 0.001 | * | RStrain_MP | -0.119 | 0.003 | * |
| Rotation_BP | -0.537 | 0.001 | * | Torsion_Basal_AS | -0.177 | 0.001 | * | LDisp_BS | -0.117 | 0.001 | * |
| RDisp_AI | -0.527 | 0.001 | * | Torsion_Regional_AA | -0.175 | 0.001 | * | Torsion_Basal_BS | -0.113 | 0.003 | * |
| RStrain_MI | -0.464 | 0.001 | * | Torsion_Regional_AI | -0.173 | 0.001 | * | Strain_3D_MI | -0.107 | 0.005 | * |
| RStrain_BL | -0.463 | 0.001 | * | Rotation_global | -0.173 | 0.001 | * | RStrain_ML | -0.105 | 0.002 | * |
| RStrain_AI | -0.423 | 0.001 | * | Twist_AS | -0.172 | 0.001 | * | RDisp_AL | -0.103 | 0.003 | * |
| LDisp_AI | -0.402 | 0.001 | * | LDisp_BS | -0.168 | 0.001 | * | CStrain_BA | -0.102 | 0.006 | * |
| RStrain_BAS | -0.397 | 0.001 | * | Strain_3D_AL | -0.160 | 0.001 | * | Torsion_Regional_BS | -0.096 | 0.013 | * |
| RStrain_BA | -0.388 | 0.001 | * | LDisp_BP | -0.150 | 0.001 | * | Rotation_MAS | -0.096 | 0.009 | * |
| RStrain_BP | -0.368 | 0.001 | * | Torsion_Basal_AA | -0.146 | 0.001 | * | Rotation_ML | -0.096 | 0.008 | * |
| RStrain_AL | -0.368 | 0.001 | * | Rotation_BAS | -0.141 | 0.001 | * | LDisp_AS | -0.095 | 0.007 | * |
| Rotation_AI | -0.363 | 0.001 | * | Torsion_Regional_MAS | -0.135 | 0.002 | * | Rotation_global | -0.094 | 0.010 | * |
| RStrain_MAS | -0.362 | 0.001 | * | Twist_AA | -0.132 | 0.002 | * | LStrain_MAS | -0.093 | 0.010 | * |
| Twist_MAS | -0.343 | 0.001 | * | Rotation_AI | -0.131 | 0.002 | * | Twist_BS | -0.093 | 0.006 | * |
| LDisp_AS | -0.339 | 0.001 | * | Torsion_Regional_MA | -0.126 | 0.002 | * | LDisp_BL | -0.089 | 0.011 | * |
| RStrain_MP | -0.315 | 0.001 | * | CStrain_MI | -0.125 | 0.002 | * | LStrain_MA | -0.087 | 0.013 | * |
| Twist_MA | -0.313 | 0.001 | * | CStrain_BA | -0.120 | 0.001 | * | Strain_3D_global | -0.086 | 0.009 | * |
| Rotation_global | -0.307 | 0.001 | * | X3DDisp_ML | -0.115 | 0.002 | * | Strain_3D_BA | -0.085 | 0.013 | * |
| RStrain_BI | -0.306 | 0.001 | * | Strain_3D_AI | -0.111 | 0.002 | * | CStrain_BP | -0.074 | 0.033 | * |
| Torsion_Basal_MAS | -0.300 | 0.001 | * | CStrain_MAS | -0.108 | 0.002 | * | LStrain_BAS | -0.069 | 0.043 | * |
| RStrain_BS | -0.292 | 0.001 | * | Torsion_Regional_MS | -0.101 | 0.003 | * | RDisp_AA | -0.067 | 0.066 |  |
| RStrain_MS | -0.292 | 0.001 | * | Rotation_AL | -0.100 | 0.008 | * | CStrain_BL | -0.067 | 0.061 |  |
| Torsion_Regional_BAS | -0.290 | 0.001 | * | Torsion_Regional_ML | -0.091 | 0.015 | * | LDisp_AA | -0.066 | 0.058 |  |
| Rotation_MA | -0.286 | 0.001 | * | Torsion_Regional_global | -0.090 | 0.012 | * | RStrain_MAS | -0.060 | 0.072 |  |
| Torsion_Basal_MA | -0.286 | 0.001 | * | LDisp_BAS | -0.089 | 0.013 | * | Strain_3D_AA | -0.058 | 0.099 |  |
| Torsion_Regional_BA | -0.283 | 0.001 | * | X3DDisp_MA | -0.083 | 0.016 | * | Rotation_MI | -0.058 | 0.112 |  |
| RStrain_ML | -0.269 | 0.001 | * | X3DDisp_MS | -0.079 | 0.032 | * | LDisp_MA | -0.058 | 0.102 |  |
| Twist_AS | -0.266 | 0.001 | * | Torsion_Basal_MA | -0.079 | 0.031 | * | X3DDisp_BA | -0.055 | 0.127 |  |
| RStrain_AA | -0.264 | 0.001 | * | RStrain_AL | -0.079 | 0.021 | * | LDisp_MAS | -0.054 | 0.123 |  |
| Torsion_Regional_ML | -0.252 | 0.001 | * | X3DDisp_MP | -0.074 | 0.047 | * | RStrain_AA | -0.054 | 0.131 |  |
| RDisp_AS | -0.252 | 0.001 | * | RStrain_MS | -0.069 | 0.055 |  | RStrain_MI | -0.050 | 0.134 |  |
| RStrain_MA | -0.250 | 0.001 | * | AreaTracking_BS | -0.069 | 0.044 | * | X3DDisp_BAS | -0.045 | 0.196 |  |
| Twist_BA | -0.240 | 0.001 | * | CStrain_MP | -0.068 | 0.056 |  | AreaTracking_BA | -0.042 | 0.227 |  |
| Twist_BAS | -0.236 | 0.001 | * | Rotation_MA | -0.066 | 0.064 |  | X3DDisp_MA | -0.037 | 0.290 |  |
| Torsion_Basal_AS | -0.235 | 0.001 | * | X3DDisp_MAS | -0.066 | 0.051 |  | AreaTracking_MA | -0.035 | 0.308 |  |
| Rotation_AL | -0.224 | 0.001 | * | AreaTracking_MA | -0.066 | 0.080 |  | RStrain_global | -0.034 | 0.329 |  |
| Torsion_Regional_AS | -0.198 | 0.001 | * | Torsion_Basal_AL | -0.062 | 0.077 |  | X3DDisp_MAS | -0.033 | 0.357 |  |
| Torsion_Basal_BAS | -0.197 | 0.001 | * | Rotation_MI | -0.059 | 0.085 |  | Volume.mL. | -0.033 | 0.339 |  |
| Torsion_Regional_MS | -0.187 | 0.001 | * | Twist_MA | -0.056 | 0.089 |  | LStrain_BA | -0.032 | 0.362 |  |
| Twist_MS | -0.187 | 0.001 | * | CStrain_BI | -0.055 | 0.124 |  | RDisp_MI | -0.031 | 0.367 |  |
| Twist_AA | -0.165 | 0.001 | * | Torsion_Regional_BA | -0.053 | 0.126 |  | LStrain_BS | -0.029 | 0.383 |  |
| Torsion_Basal_MS | -0.163 | 0.001 | * | Rotation_BS | -0.050 | 0.127 |  | CStrain_MI | -0.026 | 0.493 |  |
| Torsion_Basal_BA | -0.160 | 0.001 | * | Strain_3D_global | -0.049 | 0.164 |  | CStrain_global | -0.024 | 0.498 |  |
| Rotation_AA | -0.152 | 0.001 | * | LDisp_BL | -0.046 | 0.169 |  | Torsion_Basal_BI | -0.023 | 0.510 |  |
| Rotation_MAS | -0.140 | 0.001 | * | Strain_3D_MAS | -0.042 | 0.212 |  | Strain_3D_BS | -0.022 | 0.520 |  |
| Torsion_Basal_AA | -0.137 | 0.001 | * | LDisp_BA | -0.041 | 0.236 |  | Strain_3D_BAS | -0.022 | 0.542 |  |
| Torsion_Basal_BI | -0.121 | 0.001 | * | Rotation_MS | -0.040 | 0.271 |  | X3DDisp_AA | -0.021 | 0.546 |  |
| Torsion_Basal_AL | -0.113 | 0.002 | * | X3DDisp_MI | -0.039 | 0.255 |  | Torsion_Basal_MS | -0.016 | 0.657 |  |
| Strain_3D_BL | -0.113 | 0.006 | * | CStrain_BAS | -0.038 | 0.288 |  | RStrain_AL | -0.015 | 0.666 |  |
| Twist_AL | -0.110 | 0.002 | * | Strain_3D_BS | -0.038 | 0.277 |  | LDisp_BAS | -0.011 | 0.753 |  |
| RStrain_AS | -0.099 | 0.003 | * | Twist_AL | -0.037 | 0.276 |  | RStrain_BA | -0.008 | 0.816 |  |
| Torsion_Regional_AL | -0.095 | 0.007 | * | RStrain_MAS | -0.034 | 0.359 |  | Strain_3D_AI | -0.001 | 0.965 |  |
| Twist_global | -0.092 | 0.008 | * | AreaTracking_ML | -0.032 | 0.349 |  | Strain_3D_AL | -0.001 | 0.987 |  |
| Twist_BI | -0.084 | 0.019 | * | Twist_MAS | -0.032 | 0.362 |  | LDisp_BA | 0.001 | 0.989 |  |
| Torsion_Regional_global | -0.083 | 0.016 | * | RStrain_global | -0.028 | 0.447 |  | Twist_BAS | 0.001 | 0.974 |  |
| Torsion_Regional_BS | -0.083 | 0.025 | * | Torsion_Basal_MAS | -0.024 | 0.498 |  | Twist_BI | 0.009 | 0.823 |  |
| Torsion_Regional_BI | -0.075 | 0.033 | * | RDisp_BA | -0.024 | 0.491 |  | Myo.Vol_.mL. | 0.009 | 0.815 |  |
| Strain_3D_BAS | -0.071 | 0.042 | * | Torsion_Regional_MI | -0.023 | 0.551 |  | Torsion_Basal_BL | 0.010 | 0.761 |  |
| Torsion_Basal_global | -0.070 | 0.049 | * | RDisp_BL | -0.021 | 0.580 |  | CStrain_MS | 0.011 | 0.748 |  |
| Torsion_Regional_MP | -0.069 | 0.048 | * | LDisp_MI | -0.020 | 0.557 |  | CStrain_MA | 0.013 | 0.715 |  |
| Strain_3D_BA | -0.054 | 0.134 |  | Torsion_Basal_BA | -0.020 | 0.558 |  | Strain_3D_BI | 0.015 | 0.687 |  |
| Torsion_Regional_MA | -0.050 | 0.152 |  | Rotation_MP | -0.019 | 0.583 |  | CStrain_MP | 0.015 | 0.674 |  |
| Rotation_BA | -0.045 | 0.219 |  | RDisp_BP | -0.019 | 0.609 |  | Strain_3D_BP | 0.015 | 0.679 |  |
| Torsion_Regional_MAS | -0.040 | 0.246 |  | RDisp_BAS | -0.017 | 0.610 |  | RStrain_BP | 0.015 | 0.660 |  |
| Torsion_Regional_AA | -0.037 | 0.293 |  | X3DDisp_global | -0.016 | 0.665 |  | RDisp_MP | 0.016 | 0.653 |  |
| Strain_3D_AI | -0.030 | 0.402 |  | RStrain_BS | -0.008 | 0.840 |  | X3DDisp_BL | 0.018 | 0.601 |  |
| Twist_BS | -0.027 | 0.443 |  | Strain_3D_BAS | -0.005 | 0.872 |  | X3DDisp_AS | 0.020 | 0.532 |  |
| Strain_3D_MI | -0.019 | 0.630 |  | AreaTracking_BA | -0.004 | 0.900 |  | X3DDisp_BS | 0.021 | 0.554 |  |
| Strain_3D_AS | -0.001 | 0.979 |  | Twist_BA | -0.002 | 0.953 |  | Torsion_Basal_BAS | 0.027 | 0.439 |  |
| Torsion_Basal_BS | 0.001 | 0.970 |  | RDisp_BI | -0.001 | 0.966 |  | X3DDisp_ML | 0.028 | 0.449 |  |
| Torsion_Basal_ML | 0.008 | 0.823 |  | Rotation_BA | 0.001 | 0.980 |  | Strain_3D_BL | 0.032 | 0.393 |  |
| Rotation_AS | 0.016 | 0.670 |  | RDisp_BS | 0.002 | 0.952 |  | AreaTracking_BL | 0.032 | 0.377 |  |
| Twist_ML | 0.025 | 0.488 |  | Volume.mL. | 0.006 | 0.863 |  | RStrain_AS | 0.032 | 0.331 |  |
| Torsion_Basal_MI | 0.029 | 0.391 |  | Strain_3D_MS | 0.007 | 0.822 |  | Twist_BL | 0.034 | 0.349 |  |
| Strain_3D_BS | 0.033 | 0.358 |  | Torsion_Basal_BS | 0.014 | 0.687 |  | Strain_3D_AS | 0.035 | 0.319 |  |
| Rotation_ML | 0.048 | 0.174 |  | Torsion_Regional_BAS | 0.017 | 0.628 |  | X3DDisp_global | 0.036 | 0.304 |  |
| Strain_3D_BI | 0.050 | 0.147 |  | LDisp_global | 0.019 | 0.616 |  | LStrain_ML | 0.038 | 0.266 |  |
| Strain_3D_AL | 0.051 | 0.159 |  | RStrain_AI | 0.021 | 0.534 |  | Torsion_Regional_BL | 0.041 | 0.253 |  |
| Strain_3D_AA | 0.055 | 0.118 |  | X3DDisp_BL | 0.027 | 0.432 |  | AreaTracking_MS | 0.044 | 0.204 |  |
| Strain_3D_BP | 0.067 | 0.060 |  | Myo.Vol_.mL. | 0.028 | 0.416 |  | RStrain_BL | 0.044 | 0.206 |  |
| Twist_MI | 0.100 | 0.005 | * | Strain_3D_MA | 0.029 | 0.390 |  | RStrain_BI | 0.045 | 0.203 |  |
| Strain_3D_global | 0.105 | 0.003 | * | Torsion_Basal_BAS | 0.029 | 0.385 |  | AreaTracking_BP | 0.047 | 0.209 |  |
| Strain_3D_MS | 0.127 | 0.002 | * | AreaTracking_MP | 0.031 | 0.374 |  | RStrain_BS | 0.048 | 0.184 |  |
| Rotation_BL | 0.143 | 0.001 | * | Torsion_Basal_MS | 0.033 | 0.363 |  | Twist_MS | 0.048 | 0.159 |  |
| Strain_3D_MAS | 0.168 | 0.001 | * | X3DDisp_BA | 0.034 | 0.355 |  | RStrain_BAS | 0.049 | 0.170 |  |
| Rotation_BAS | 0.173 | 0.001 | * | RDisp_MA | 0.034 | 0.317 |  | Torsion_Regional_BAS | 0.056 | 0.107 |  |
| Strain_3D_MP | 0.180 | 0.001 | * | Twist_BAS | 0.036 | 0.276 |  | Strain_3D_MS | 0.059 | 0.087 |  |
| Torsion_Regional_BL | 0.189 | 0.001 | * | Twist_MS | 0.036 | 0.303 |  | RDisp_MS | 0.063 | 0.065 |  |
| Torsion_Basal_AI | 0.199 | 0.001 | * | AreaTracking_BAS | 0.037 | 0.293 |  | LStrain_MS | 0.067 | 0.065 |  |
| Strain_3D_ML | 0.216 | 0.001 | * | Torsion_Regional_BS | 0.040 | 0.254 |  | AreaTracking_ML | 0.067 | 0.054 |  |
| Torsion_Basal_MP | 0.226 | 0.001 | * | Twist_BS | 0.044 | 0.230 |  | X3DDisp_AL | 0.068 | 0.057 |  |
| Twist_AI | 0.237 | 0.001 | * | RStrain_MI | 0.045 | 0.166 |  | AreaTracking_global | 0.080 | 0.033 | * |
| Torsion_Regional_MI | 0.253 | 0.001 | * | Strain_3D_BA | 0.046 | 0.187 |  | X3DDisp_MS | 0.082 | 0.016 | * |
| Strain_3D_MA | 0.256 | 0.001 | * | LDisp_MP | 0.051 | 0.153 |  | LStrain_BI | 0.086 | 0.011 | * |
| Twist_MP | 0.260 | 0.001 | * | RStrain_BL | 0.056 | 0.103 |  | CStrain_ML | 0.087 | 0.014 | * |
| Torsion_Basal_BP | 0.298 | 0.001 | * | Torsion_Basal_AI | 0.059 | 0.102 |  | Torsion_Regional_BI | 0.090 | 0.015 | * |
| Torsion_Regional_BP | 0.306 | 0.001 | * | AreaTracking_MS | 0.059 | 0.096 |  | RDisp_ML | 0.091 | 0.012 | * |
| Torsion_Regional_AI | 0.338 | 0.001 | * | RDisp_ML | 0.060 | 0.100 |  | Torsion_Basal_BA | 0.092 | 0.010 | * |
| Twist_BP | 0.348 | 0.001 | * | Twist_global | 0.064 | 0.070 |  | Twist_BA | 0.096 | 0.006 | * |
| Twist_BL | 0.356 | 0.001 | * | RDisp_MP | 0.064 | 0.061 |  | X3DDisp_BI | 0.100 | 0.008 | * |
| Rotation_MS | 0.360 | 0.001 | * | AreaTracking_MAS | 0.070 | 0.041 | * | LStrain_AA | 0.103 | 0.006 | * |
| Torsion_Basal_BL | 0.363 | 0.001 | * | LDisp_MS | 0.077 | 0.027 | * | LStrain_global | 0.110 | 0.006 | * |
| CStrain_BI | 0.403 | 0.001 | * | Torsion_Regional_MP | 0.081 | 0.019 | * | CStrain_AA | 0.111 | 0.005 | * |
| CStrain_BS | 0.418 | 0.001 | * | X3DDisp_BAS | 0.081 | 0.023 | * | X3DDisp_AI | 0.112 | 0.003 | * |
| Rotation_BS | 0.490 | 0.001 | * | LStrain_global | 0.082 | 0.019 | * | RStrain_MS | 0.112 | 0.002 | * |
| LStrain_BL | 0.501 | 0.001 | * | CStrain_BP | 0.084 | 0.020 | * | AreaTracking_MP | 0.112 | 0.003 | * |
| LStrain_BA | 0.523 | 0.001 | * | Strain_3D_MI | 0.088 | 0.011 | * | X3DDisp_MP | 0.115 | 0.003 | * |
| LStrain_AS | 0.533 | 0.001 | * | RDisp_MI | 0.088 | 0.010 | * | X3DDisp_BP | 0.118 | 0.002 | * |
| AreaTracking_AS | 0.569 | 0.001 | * | AreaTracking_BI | 0.089 | 0.015 | * | Torsion_Basal_MAS | 0.123 | 0.002 | * |
| CStrain_MI | 0.595 | 0.001 | * | RDisp_global | 0.089 | 0.014 | * | Rotation_AS | 0.127 | 0.001 | * |
| CStrain_AI | 0.611 | 0.001 | * | Strain_3D_BL | 0.092 | 0.005 | * | RDisp_global | 0.134 | 0.001 | * |
| AreaTracking_AI | 0.624 | 0.001 | * | Rotation_BL | 0.096 | 0.007 | * | Rotation_MA | 0.134 | 0.001 | * |
| LStrain_AI | 0.651 | 0.001 | * | Strain_3D_BP | 0.098 | 0.007 | * | Twist_MAS | 0.135 | 0.001 | * |
| CStrain_AS | 0.651 | 0.001 | * | CStrain_BL | 0.099 | 0.008 | * | Torsion_Regional_AI | 0.136 | 0.002 | * |
| CStrain_MS | 0.660 | 0.001 | * | Torsion_Basal_global | 0.101 | 0.005 | * | Torsion_Regional_AA | 0.138 | 0.001 | * |
| AreaTracking_BS | 0.666 | 0.001 | * | Twist_AI | 0.102 | 0.006 | * | CStrain_AS | 0.145 | 0.001 | * |
| AreaTracking_AL | 0.666 | 0.001 | * | X3DDisp_BP | 0.112 | 0.002 | * | AreaTracking_AA | 0.146 | 0.001 | * |
| AreaTracking_BI | 0.668 | 0.001 | * | Strain_3D_ML | 0.114 | 0.002 | * | X3DDisp_MI | 0.150 | 0.001 | * |
| CStrain_AA | 0.691 | 0.001 | * | LDisp_MAS | 0.117 | 0.002 | * | AreaTracking_MI | 0.151 | 0.001 | * |
| X3DDisp_AS | 0.703 | 0.001 | * | RStrain_BP | 0.117 | 0.003 | * | LStrain_AL | 0.151 | 0.001 | * |
| AreaTracking_AA | 0.703 | 0.001 | * | AreaTracking_MI | 0.117 | 0.001 | * | Torsion_Regional_AL | 0.151 | 0.001 | * |
| LStrain_BI | 0.710 | 0.001 | * | RDisp_MAS | 0.118 | 0.002 | * | Torsion_Basal_BP | 0.151 | 0.001 | * |
| CStrain_AL | 0.715 | 0.001 | * | Torsion_Basal_ML | 0.121 | 0.001 | * | RDisp_MA | 0.158 | 0.001 | * |
| LStrain_BS | 0.727 | 0.001 | * | Strain_3D_MP | 0.124 | 0.002 | * | Twist_BP | 0.161 | 0.001 | * |
| LStrain_BP | 0.734 | 0.001 | * | X3DDisp_BS | 0.126 | 0.002 | * | RDisp_BP | 0.166 | 0.001 | * |
| X3DDisp_AI | 0.749 | 0.001 | * | RStrain_BAS | 0.130 | 0.001 | * | Torsion_Regional_BA | 0.167 | 0.001 | * |
| LStrain_BAS | 0.768 | 0.001 | * | LDisp_MA | 0.133 | 0.001 | * | RDisp_BL | 0.174 | 0.001 | * |
| AreaTracking_BA | 0.770 | 0.001 | * | Strain_3D_BI | 0.135 | 0.001 | * | AreaTracking_AS | 0.180 | 0.001 | * |
| CStrain_BA | 0.786 | 0.001 | * | RStrain_MA | 0.137 | 0.001 | * | Rotation_AI | 0.186 | 0.001 | * |
| CStrain_MA | 0.786 | 0.001 | * | RStrain_BA | 0.137 | 0.002 | * | Rotation_AL | 0.186 | 0.001 | * |
| Myo.Vol_.mL. | 0.803 | 0.001 | * | Twist_ML | 0.143 | 0.001 | * | LStrain_BL | 0.191 | 0.001 | * |
| AreaTracking_BL | 0.809 | 0.001 | * | LDisp_ML | 0.147 | 0.001 | * | Torsion_Basal_AS | 0.193 | 0.001 | * |
| X3DDisp_AA | 0.812 | 0.001 | * | RStrain_MP | 0.149 | 0.001 | * | LStrain_MP | 0.198 | 0.001 | * |
| X3DDisp_AL | 0.821 | 0.001 | * | Rotation_ML | 0.153 | 0.001 | * | Torsion_Regional_BP | 0.207 | 0.001 | * |
| CStrain_ML | 0.823 | 0.001 | * | LStrain_ML | 0.155 | 0.001 | * | LStrain_BP | 0.208 | 0.001 | * |
| LStrain_AA | 0.826 | 0.001 | * | X3DDisp_BI | 0.155 | 0.001 | * | Torsion_Regional_MS | 0.210 | 0.001 | * |
| CStrain_BAS | 0.827 | 0.001 | * | RStrain_ML | 0.166 | 0.001 | * | LStrain_MI | 0.214 | 0.001 | * |
| CStrain_BL | 0.844 | 0.001 | * | LStrain_MA | 0.185 | 0.001 | * | RDisp_MAS | 0.218 | 0.001 | * |
| AreaTracking_MI | 0.846 | 0.001 | * | AreaTracking_BL | 0.186 | 0.001 | * | CStrain_AL | 0.229 | 0.001 | * |
| LStrain_AL | 0.849 | 0.001 | * | LStrain_BS | 0.186 | 0.001 | * | LStrain_AS | 0.230 | 0.001 | * |
| AreaTracking_BAS | 0.857 | 0.001 | * | AreaTracking_BP | 0.186 | 0.001 | * | RDisp_BA | 0.231 | 0.001 | * |
| X3DDisp_MS | 0.864 | 0.001 | * | LStrain_BAS | 0.187 | 0.001 | * | Twist_AS | 0.237 | 0.001 | * |
| CStrain_MAS | 0.874 | 0.001 | * | LStrain_MP | 0.188 | 0.001 | * | AreaTracking_AL | 0.241 | 0.001 | * |
| X3DDisp_MI | 0.881 | 0.001 | * | RStrain_BI | 0.189 | 0.001 | * | CStrain_AI | 0.241 | 0.001 | * |
| AreaTracking_BP | 0.882 | 0.001 | * | Torsion_Regional_BL | 0.193 | 0.001 | * | Torsion_Basal_MI | 0.246 | 0.001 | * |
| LStrain_MI | 0.883 | 0.001 | * | RDisp_MS | 0.198 | 0.001 | * | RDisp_BI | 0.246 | 0.001 | * |
| CStrain_BP | 0.885 | 0.001 | * | Torsion_Basal_BL | 0.220 | 0.001 | * | Torsion_Basal_ML | 0.254 | 0.001 | * |
| AreaTracking_MS | 0.885 | 0.001 | * | LStrain_MAS | 0.225 | 0.001 | * | Torsion_Basal_global | 0.266 | 0.001 | * |
| AreaTracking_ML | 0.908 | 0.001 | * | LStrain_MS | 0.229 | 0.001 | * | Rotation_AA | 0.269 | 0.001 | * |
| X3DDisp_MAS | 0.910 | 0.001 | * | RDisp_AL | 0.242 | 0.001 | * | Torsion_Regional_MAS | 0.275 | 0.001 | * |
| X3DDisp_MP | 0.910 | 0.001 | * | Twist_BL | 0.251 | 0.001 | * | Torsion_Regional_AS | 0.275 | 0.001 | * |
| CStrain_MP | 0.910 | 0.001 | * | LStrain_BI | 0.252 | 0.001 | * | Torsion_Basal_MA | 0.283 | 0.001 | * |
| Volume.mL. | 0.911 | 0.001 | * | Torsion_Basal_BP | 0.261 | 0.001 | * | Twist_MI | 0.285 | 0.001 | * |
| AreaTracking_MA | 0.911 | 0.001 | * | Torsion_Basal_MI | 0.261 | 0.001 | * | Twist_ML | 0.287 | 0.001 | * |
| LStrain_ML | 0.916 | 0.001 | * | Twist_BI | 0.265 | 0.001 | * | AreaTracking_AI | 0.290 | 0.001 | * |
| LStrain_MP | 0.917 | 0.001 | * | RDisp_AA | 0.265 | 0.001 | * | RDisp_BS | 0.301 | 0.001 | * |
| LStrain_MS | 0.921 | 0.001 | * | Twist_MI | 0.267 | 0.001 | * | Twist_MA | 0.304 | 0.001 | * |
| LStrain_MA | 0.922 | 0.001 | * | LStrain_MI | 0.270 | 0.001 | * | LStrain_AI | 0.321 | 0.001 | * |
| X3DDisp_ML | 0.925 | 0.001 | * | Torsion_Basal_MP | 0.272 | 0.001 | * | RDisp_BAS | 0.344 | 0.001 | * |
| AreaTracking_MAS | 0.926 | 0.001 | * | Torsion_Basal_BI | 0.279 | 0.001 | * | Torsion_Basal_AA | 0.348 | 0.001 | * |
| LStrain_MAS | 0.927 | 0.001 | * | Torsion_Regional_BI | 0.285 | 0.001 | * | Twist_AA | 0.352 | 0.001 | * |
| X3DDisp_BI | 0.927 | 0.001 | * | Torsion_Regional_BP | 0.287 | 0.001 | * | Twist_global | 0.365 | 0.001 | * |
| X3DDisp_MA | 0.929 | 0.001 | * | Twist_BP | 0.308 | 0.001 | * | Torsion_Basal_MP | 0.369 | 0.001 | * |
| AreaTracking_MP | 0.938 | 0.001 | * | RDisp_AS | 0.311 | 0.001 | * | Twist_MP | 0.376 | 0.001 | * |
| X3DDisp_BP | 0.939 | 0.001 | * | RDisp_AI | 0.316 | 0.001 | * | Torsion_Regional_MA | 0.379 | 0.001 | * |
| CStrain_global | 0.940 | 0.001 | * | Twist_MP | 0.325 | 0.001 | * | Torsion_Basal_AL | 0.403 | 0.001 | * |
| X3DDisp_BS | 0.941 | 0.001 | * | LStrain_BP | 0.325 | 0.001 | * | Torsion_Basal_AI | 0.408 | 0.001 | * |
| X3DDisp_BA | 0.949 | 0.001 | * | LStrain_BA | 0.330 | 0.001 | * | Twist_AL | 0.410 | 0.001 | * |
| X3DDisp_BL | 0.949 | 0.001 | * | LStrain_BL | 0.339 | 0.001 | * | Torsion_Regional_global | 0.413 | 0.001 | * |
| AreaTracking_global | 0.950 | 0.001 | * | LDisp_AL | 0.398 | 0.001 | * | Twist_AI | 0.417 | 0.001 | * |
| X3DDisp_global | 0.951 | 0.001 | * | LDisp_AI | 0.432 | 0.001 | * | Torsion_Regional_MI | 0.421 | 0.001 | * |
| X3DDisp_BAS | 0.952 | 0.001 | * | LDisp_AA | 0.474 | 0.001 | * | Torsion_Regional_ML | 0.422 | 0.001 | * |
| LStrain_global | 0.972 | 0.001 | * | LDisp_AS | 0.526 | 0.001 | * | Torsion_Regional_MP | 0.489 | 0.001 | * |

**Title page**

Left Atrial trajectory impairment in Hypertrophic Cardiomyopathy disclosed by Geometric Morphometrics and Parallel Transport

Paolo Piras^1,2^

Concetta Torromeo^2^

Federica Re^3^

Antonietta Evangelista^4^

Stefano Gabriele^5^

Giuseppe Esposito^2^

Paola Nardinocchi^1^

Luciano Teresi^6^

Andrea Madeo^2,3^

Claudia Chialastri^3^

Michele Schiariti^2^

Valerio Varano^5^

Massimo Uguccioni^3^

Paolo E. Puddu^1^

^1^Dipartimento di Ingegneria Strutturale e Geotecnica, Sapienza Università di Roma, Roma, Italy;

^2^Dipartimento di Scienze Cardiovascolari, Respiratorie, Nefrologiche, Anestesiologiche e Geriatriche, Sapienza Università di Roma, Roma, Italy;

^3^Centro per le Cardiomiopatie Ospedale S. Camillo-Forlanini, Roma, Italy;

^4^Ospedale San Giovanni Calibita Fatebenefratelli Isola Tiberina, Roma, Italy;

^5^Dipartimento di Architettura, Università Roma Tre, Roma, Italy

^6^Dipartimento di Matematica e Fisica, LaMS-Modeling & Simulation Lab, Università Roma Tre, Roma, Italy.

**Supplementary video legend**: Animation corresponding to Figure 4 in the main manuscript. Differential LA deformation patterns in Controls and HCM. LA end-diastolic and end-systolic states for both Control and HCM subjects are shown. It is evident that in Controls we see a stronger contraction and deformation at end-diastole relatively to HCM. For the sake of simplicity colormap refers, here, to the deformation relative to end-systolic state.
